# Supplementary material for: A single-cell analysis of breast cancer cell lines to study tumour heterogeneity and drug response
Source: Nat Commun. 2022 Mar 31;13:1714. doi: 10.1038/s41467-022-29358-6 (PMC8971486; doi:10.1038/s41467-022-29358-6)
Supplement: Supplementary file 1 — Supplementary Information [file 41467_2022_29358_MOESM1_ESM.pdf]

A single-cell atlas of breast cancer cell lines to study tumour heterogeneity and drug response.

AUTHORS

Gambardella G[1,2,*], Viscido G[1,2,*], Tumaini B[1], Isacchi A[3], Bosotti R[3], di Bernardo D.

AFFILIATIONS

[1]Telethon Institute of Genetics and Medicine, Naples, Italy

[2]University of Naples Federico II, Department of Chemical Materials and Industrial Engineering, Naples, Italy

[3]NMSsrl, Nerviano Medical Sciences, 20014, Nerviano, Milan, Italy

CORRESPONDENCE:
dibernardo@tigem.it

***These authors contributed equally to this work.**

Table Contents

Supplementary Note 1. 2

Supplementary Figures. 3

Supplementary Tables.. 27

Supplementary Note 1.

The model is depicted in Supplementary Figure 13 and it assumes that each cell can be in either one of two states (Her2- and Her2+) and can switch dynamically between the two with rates λ, δ. Moreover, independently of the state, the cell can replicate with rates k_1 and k_2. Finally, the effect of anti-Her2 drugs is present as an additional degradation term on Her2+ cells.

Using standard mass action kinetics, the following equations describing the model in Supplementary Figure 13 can be derived:

$$\begin{cases} \dot{h^-} = (k_1 - \lambda)h^- + \delta h^+ \\ \dot{h^+} = (k_2 - \delta)h^+ + \lambda h^- - u h^+ \end{cases}$$

Where h^- stands for Her2$^-$ cells, and h^+ for Her2$^+$ cells, whereas u quantifies the effect of anti-Her2 drugs (e.g., Afatinib). To simplify the model, the replication rates k1 and k2 are assumed to be the same. The parameters values for λ, δ determine the percentage of Her2$^+$ cells in the cell population, which can be shown to be equal to $\frac{\lambda}{\lambda+\delta}$ after a transient, when no drug is present (u=0). The parameters' values, reported in Supplementary Figure 13, were set to yield a doubling rate of the total cell population ($h^- + h^+$) of approx. 3.5 days, like the observed cell cycle rate of the MDAMB361 cell line, and a percentage of Her+ cells of 60%, close to the experimentally measured value reported in Figure 3B of the main manuscript. With these nominal values, the replication rates and the interconversion rates are of the same order of magnitude.

Numerical simulations of the model behaviour following treatment with Afatinib are reported in Supplementary Figure 23 with a starting population of $1x10^6$ cells, of which $0.9x10^6$ are Her2+ cells and $0.1x10^6$ Her2- cells (i.e. 90% Her2+ cells): for nominal values of the parameters, in the absence of Afatinib, both Her2- and Her2+ cells grow exponentially, while the percentage of Her2+ cells quickly stabilises at 60%; upon Afatinib treatment for 3 days, both the number of Her2+ and Her 2- cells decrease, while the percentage of Her2+ cells settles at 30%. Finally, upon removal of Afatinib, the number of cells increases while the percentage of Her2+ cells recover to 60%. When the interconversion rates (λ, δ) are much slower than the growth rate (k), then in the absence of Afatinib the percentage of Her2+ cells take longer to stabilise at 60%, whereas the effect of a 3 days Afatinib is much more pronounced, causing the percentage of Her2 cells to quickly drop to approx. 10%. This can be explained by the fact that Her2- cells keep increasing in number during Afatinib treatment as their growth rate is much faster than their interconversion rate, while Her2+ are removed by Afatinib treatment and cannot escape its effect as they convert to Her2- cells too slowly. Upon Afatinib removal, both the number of cells and the percentage of Her2+ cells start increasing. For fast interconversion rates, the situation is reversed, that is cells increase in number in the absence of Afatinib with the percentage of Her2+ cells quickly reaching 60%. Interestingly, while the effect of Afatinib is almost absent in terms of changes in the percentage of Her2% cells, the total number of cells drops substantially, as Her2- are much more affected by Afatinib treatment because of their fast interconversion to Her2+ cells.

We also simulated dose response curves at increasing concentrations of drugs (i.e., the value of u in the model) for the model for each set of parameters' values (slow, nominal and fast), as reported in Supplementary Figure 24. As expected, only in the case of slow interconversion, it is possible to appreciate a difference in the response of Her2- cells versus Her2+ cells following treatment with Afatinib.

The experimental data presented in Figure 3B of the main manuscript are all compatible with the modelling results when using the nominal parameters, that is the interconversion rate is of the same order of magnitude as the growth rate (i.e., cell cycle).

Supplementary Figures.

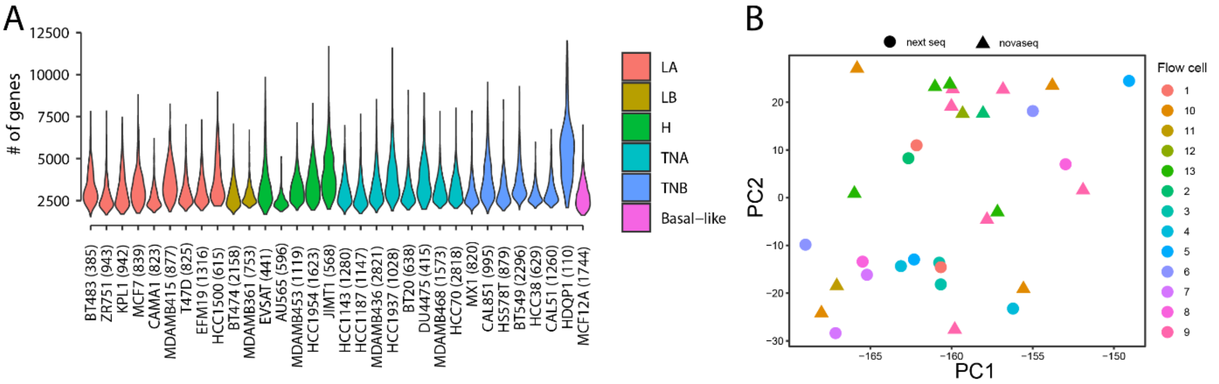

Supplementary Figure 01 – Distribution of the number of captured genes per cell across cell-lines and batch effect estimation. (A) Each violin plot represents the distribution of the number of captured genes per cell in the indicated cell-line on the x-axis, where the number between parenthesis indicates the number of sequenced cells. (**B**) In the PCA plot each cell line is represented by a dot after conversion in pseudo-bulk (Methods), whose colour represents the batch (i.e. the cell lines sequenced in the same flow cell) and whose shape, the sequencing platform (i.e. Illumina Novaeq 6000 or NextSeq500). Source data are provided in a Source data file.

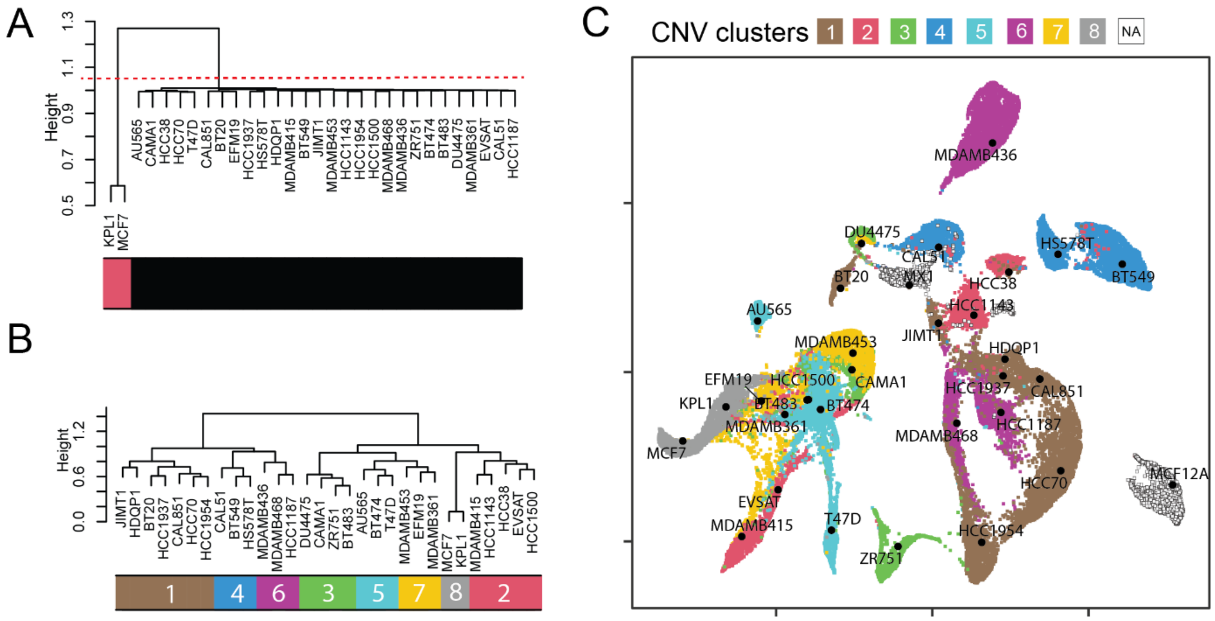

Supplementary Figure 02 – Relationship between genomic features of cell lines and single-cell clusters. (**A**) Hierarchical clustering of 30 out of 32 BC cell lines according to the number of genomic variants they share. (**B**) Hierarchical clustering of 30 out of 32 BC cell lines according to their Copy Number Alteration (CNV) profiles. (**C**) The clusters identified in panel B are superimposed onto the single cell BC atlas. Cell line genomic variants and CNV profiles of 30 (out of 32) BC cell lines were available in the COSMIC repository.

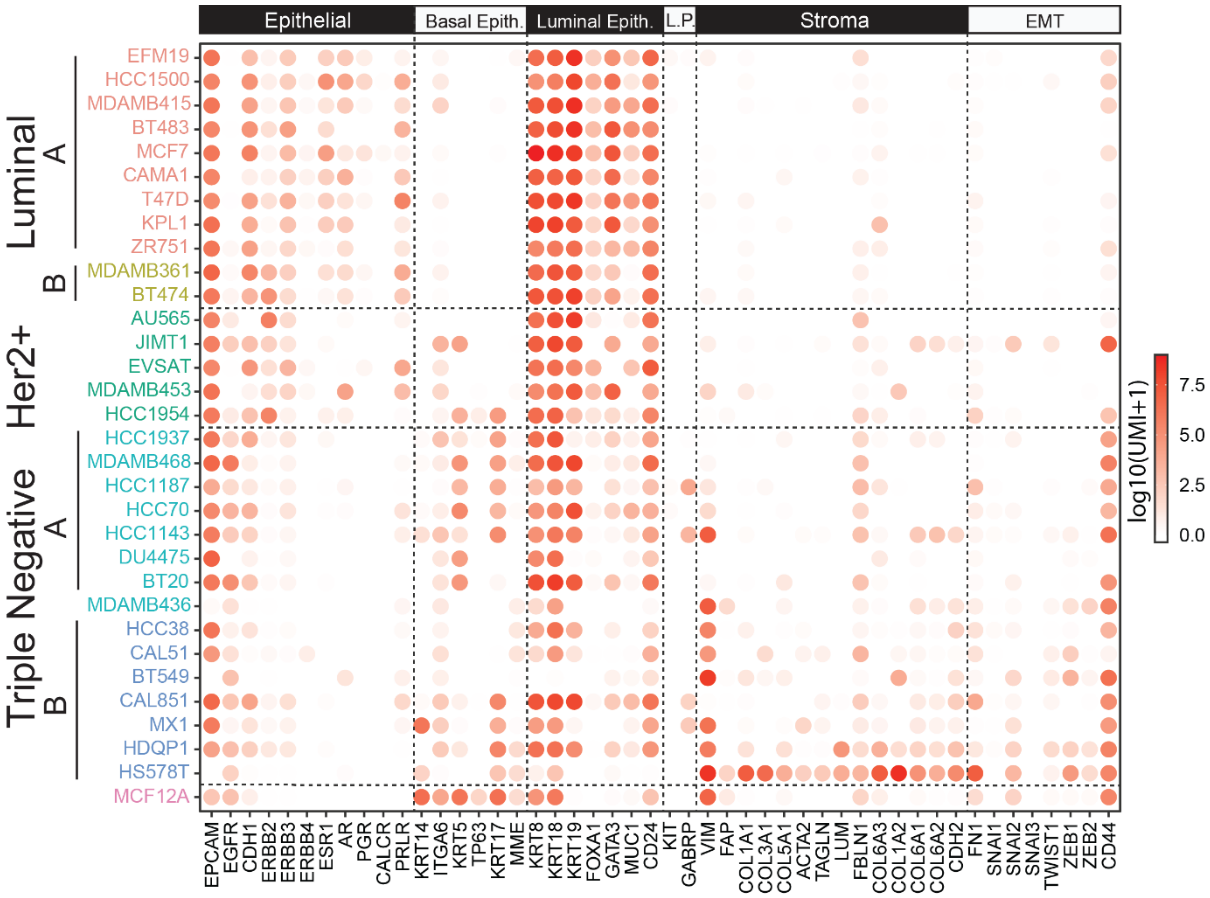

Supplementary Figure 03 – Average expression of literature-based biomarker genes across the 32 sequenced BC cell lines. Dotplot of literature-based biomarker genes along the columns for each of the 32 sequenced cell lines along the rows. Biomarker genes are grouped by type (Basal Epith. = Basal Epithelial, Luminal Epith. = Luminal Epithelial, L.P. = Luminal Progenitor, EMT = Epithelial to Mesenchymal Transition). Source data are provided in a Source data file.

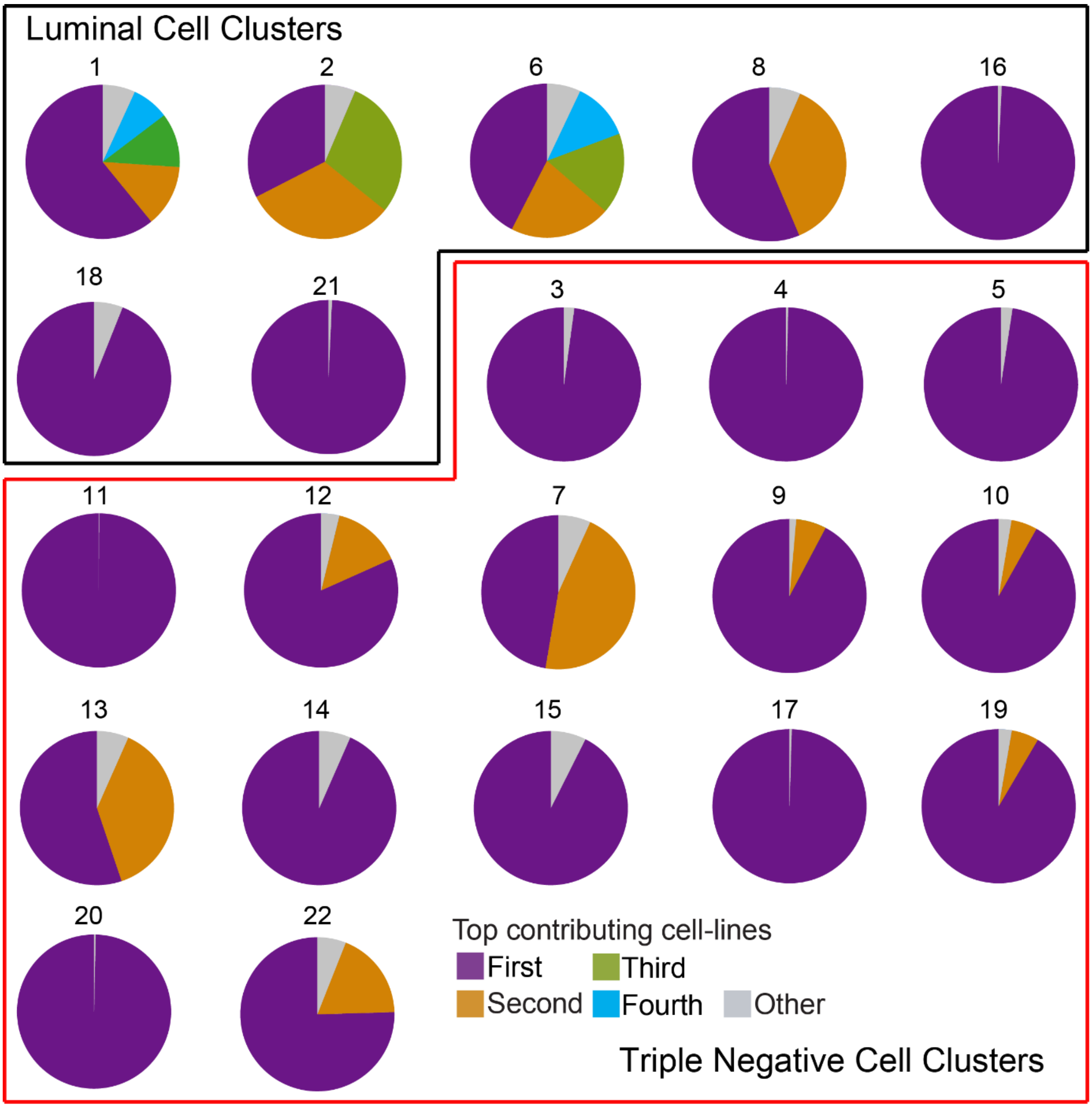

Supplementary Figure 04: Composition of the clusters in the atlas. For the indicated cluster, the corresponding pie-chart represents the cluster composition in terms of cell lines. Cell-lines in the same pie-chart are distinguished by colour. Gray represents the cell-lines in the cluster contributing with less than 5% of total cells in the cluster, while the other colours represent distinct cell lines. Source data are provided in a Source data file.

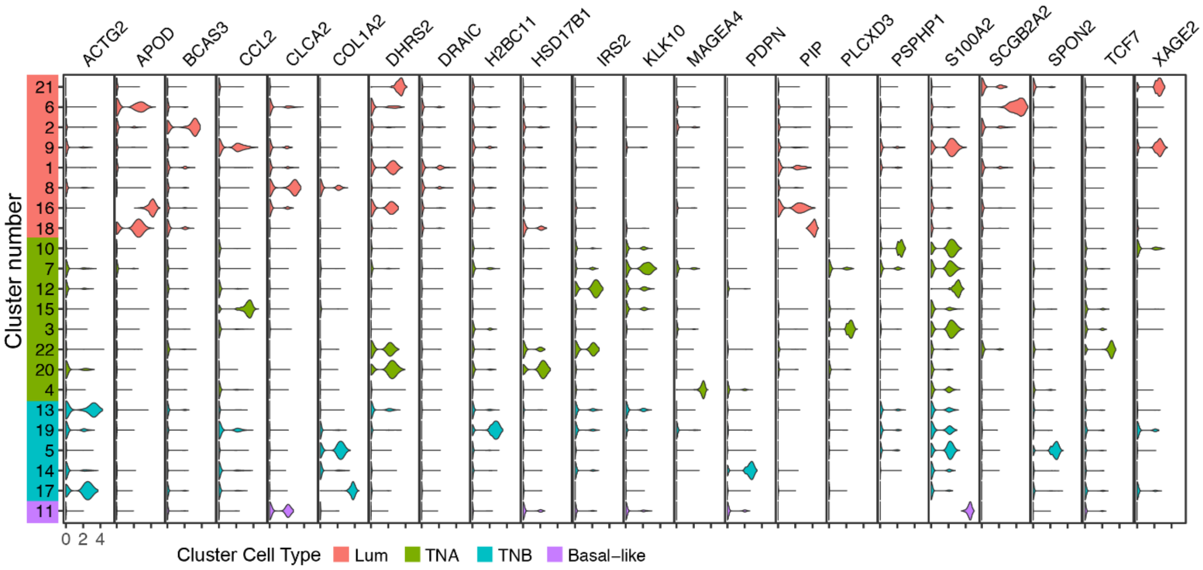

Supplementary Figure 05 – Expression distribution across the 22 clusters of the 22 biomarker genes. Biomarker genes, one for each cluster, were identified by selecting the most differentially expressed gene in each cluster. Clusters on the y-axis are color-coded according to the breast cancer subtype of majority of cells they are composed of (Lum: Luminal A and B; TNA: Triple-Negative A; TNB: Triple Negative B).

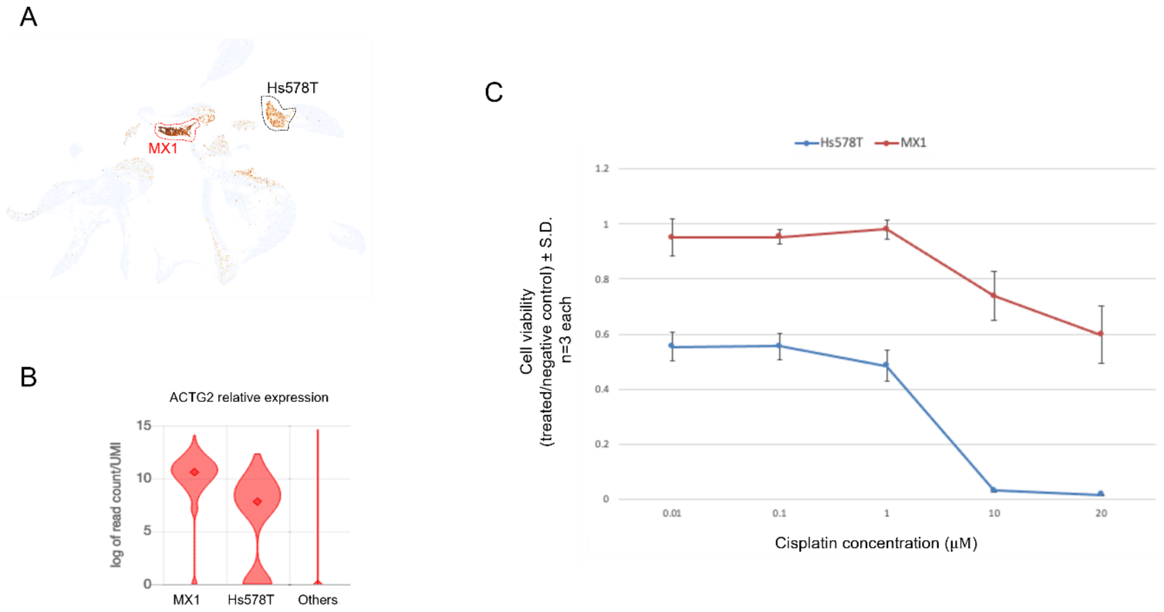

Supplementary Figure 06 – Correlation between ACTG2 expression and response to cisplatin. (**A**) Expression of ACTG2 gene across the 35,276 sequenced cells. (**B**) Violin plot showing the expression distribution of ACTG2 in MX1 and Hs578t cell lines against the other sequenced cell lines in the single-cell BC atlas. Median value is indicated as a point in the violin plot. Source data are provided in a Source data file. (**C**) Dose response curve measuring cell viability (y-axis) following treatment with Cisplatin for 72h at the indicated concentrations in the MX1 cell line (red line) and the Hs578t cell line (blue line). For each point in **C**, n=3. Source data are provided in a Source data file.

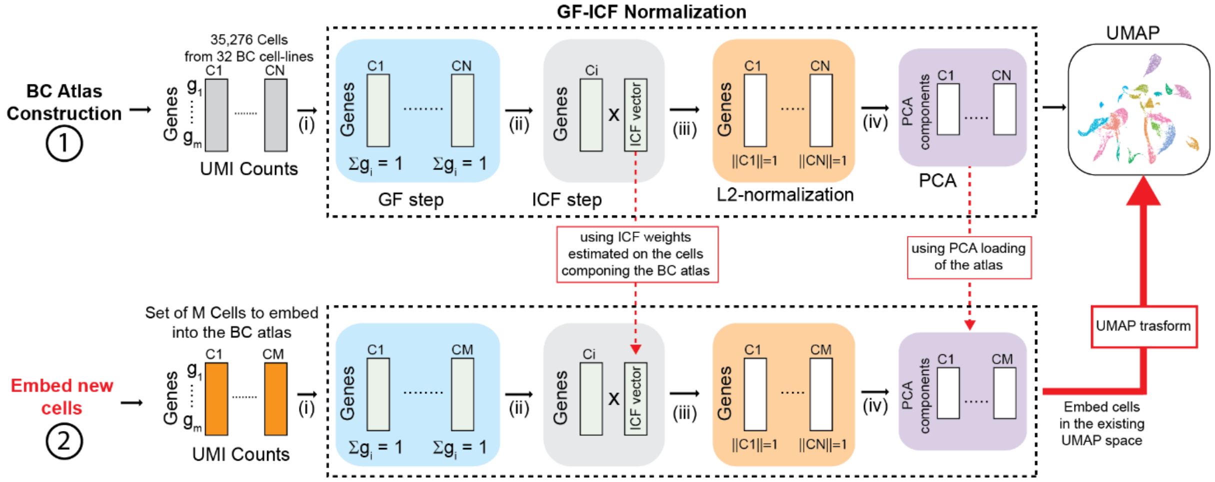

Supplementary Figure 07- Description of the single-cell mapping algorithm to add new single cell transcriptional profiles to the BC atlas. Following BC atlas construction (step 1), additional scRNA-seq profiles (or tiles from 10x spatial transcriptomics dataset) can be added to the atlas (step 2) by first normalizing scRNA-seq data with the *gficf* package using the ICF weights estimated during the BC atlas construction and then by projecting the normalised scRNA-seq to the Principal Component space using gene loadings from the BC atlas. Finally, the *umap_transform* function of *uwot* package in R, which uses the UMAP estimated model, is used to embed the new cells into the existing UMAP space.

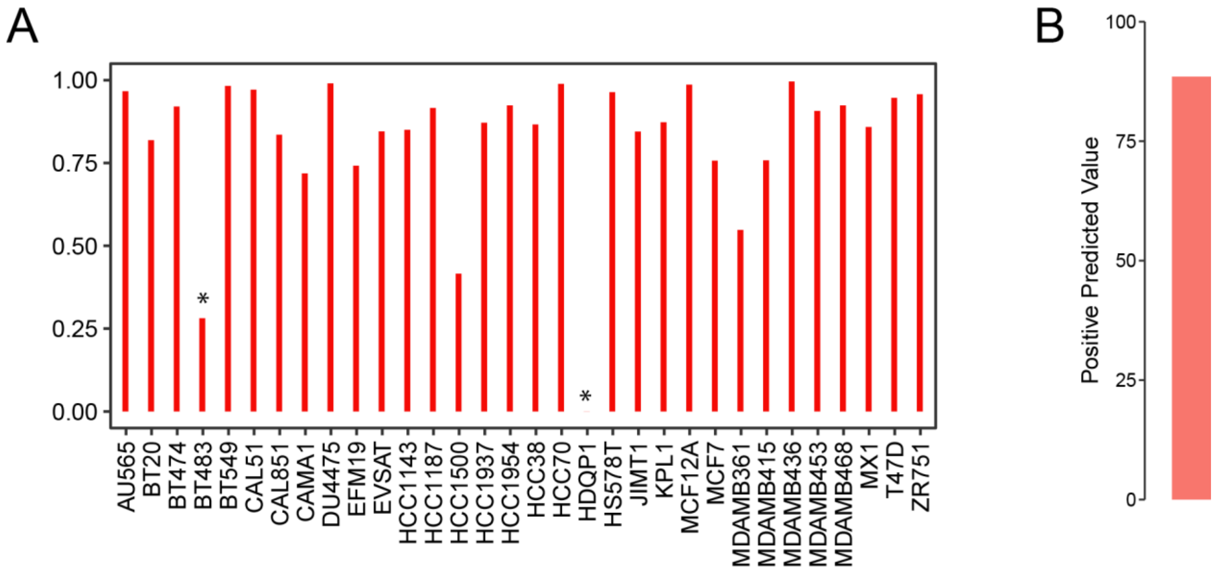

Supplementary Figure 08 – Validation of the single-cell mapping algorithm. (**A**) Positive Predictive Value (PPV) of the mapping algorithm in correctly mapping the cell line of origin from single cell transcriptional profiles divided by cell-lines. The algorithm was first trained on the single-cell transcriptional profiles of 75% of the cells in each cell-line and then its performance was tested on the remaining 25% of the cells. Details can be found in the Methods section of the main text. The asterisk indicates cell lines with the lowest number of sequenced cells in the dataset, i.e., HDQP1 with 110 cells and BT483 with 385 cell. (**B**) Overall performance of the mapping algorithm in correctly classifying the cell line of origin from single cell transcriptional profiles. In this case the PPV is estimated considering all the cells of the test set. Source data are provided in a Source data file.

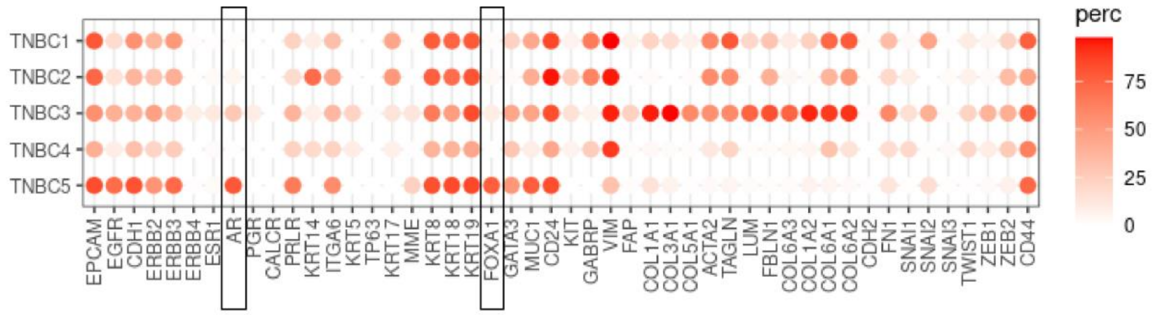

Supplementary Figure 09 – Percentage of cells expressing literature-based biomarker genes across the 5 TNBC patients. Dotplot of literature-based biomarker genes along the columns for each of the TNBC patients whose data are available from Gao et al, Nature Biotechnology, 2021 (reference 50 in the main text). Source data are provided in a Source data file.

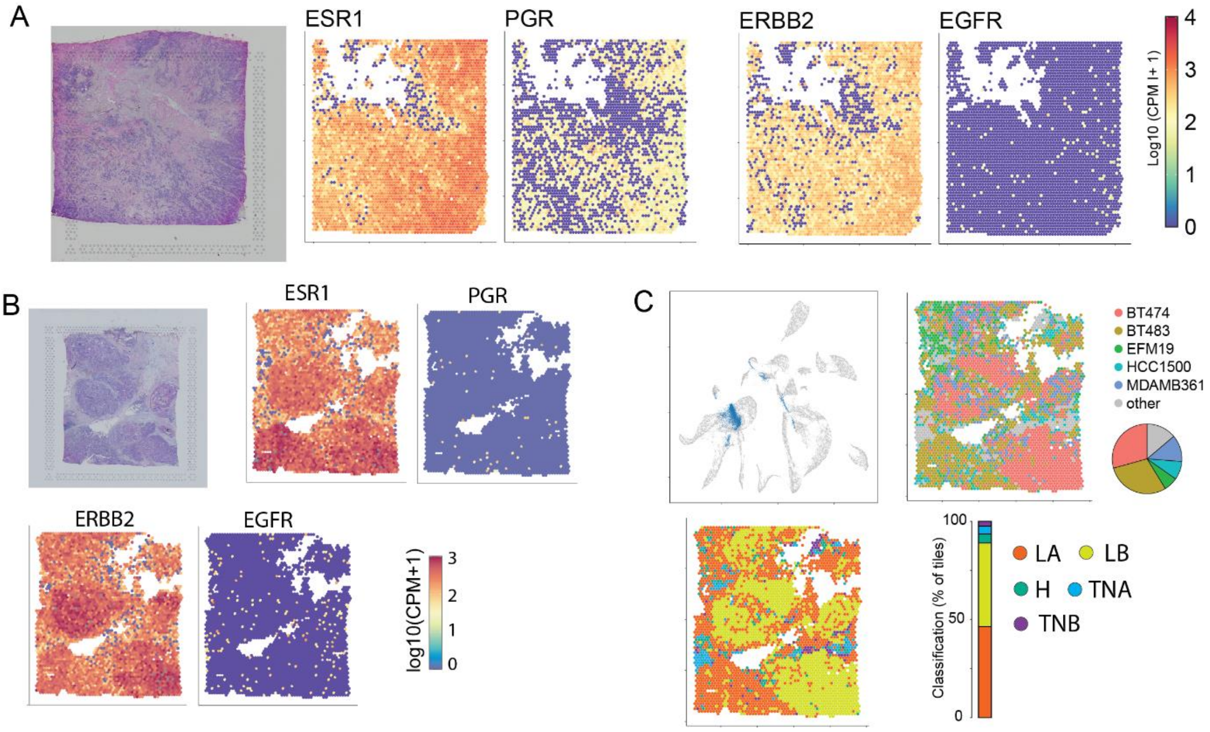

Supplementary Figure 10 – Automatic detection of cell line composition of spatial transcriptomics profiles . (A) Tissue-slide of the lobular BC tumour biopsy (presented in Figure 2C) analysed by means of 10x Genomics Visium spatial transcriptomics with spatial expression of ESR1, PGR, ERBB2 and EGFR genes. (**B**) Tissue-slide of a ductal BC tumour biopsy analysed by means of 10x Genomics Visium spatial transcriptomics with the spatial expression of ESR1, PGR, ERBB2 and EGFR genes. (**C**) Top-left: The RNA-seq of spatial tiles of the patient in panel B were embedded in the BC atlas to predict which cell-line they are most similar to with the mapping algorithm described in the main text. Top-right: Classification of each spatial tile in terms of cell-lines in the spatial context with the pie-chart reporting the percentage of spatial tiles predicted to be similar a specific cell-line. Bottom: Classification of each spatial tile in terms of tumour type in the spatial context. Bottom-right: The stacked bar plot reports the percentage of spatial tiles according to the predicted tumour type. All spatial transcriptomics data were downloaded from the 10X Genomics website.

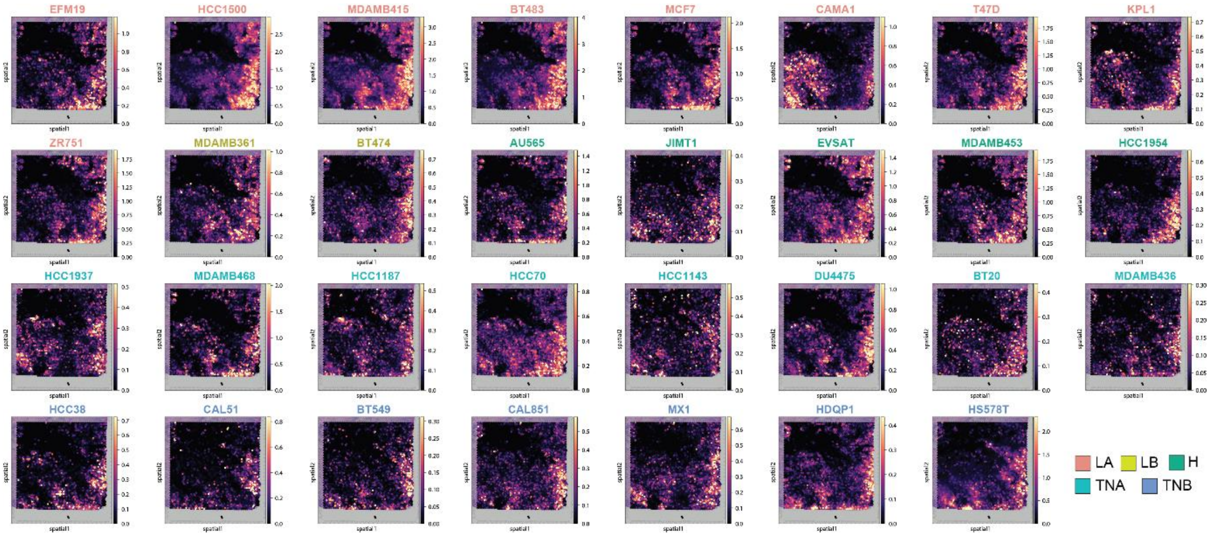

Supplementary Figure 11 – Cell line compostion from spatial transcriptomic data of the lobular BC patient shown in Figure 2C using the Cell2Location algorithm to deconvolve each spatial tile to multiple cell lines. Cell2Location cell-line is applied to the transcriptomic data of each spatial tile to estimate its cell line composition using the BC single cell atlas. The relative abundance of each of the 31 BC cell lines estimated from Cell2Location across the tissue biopsie is represented on a colour scale with brighter spots reporting highest abundance. for the lobular BC patient for which spatial transcriptomic data was available from 10x Genomics repository. The name of each cell-line is coloured according to its BC type.

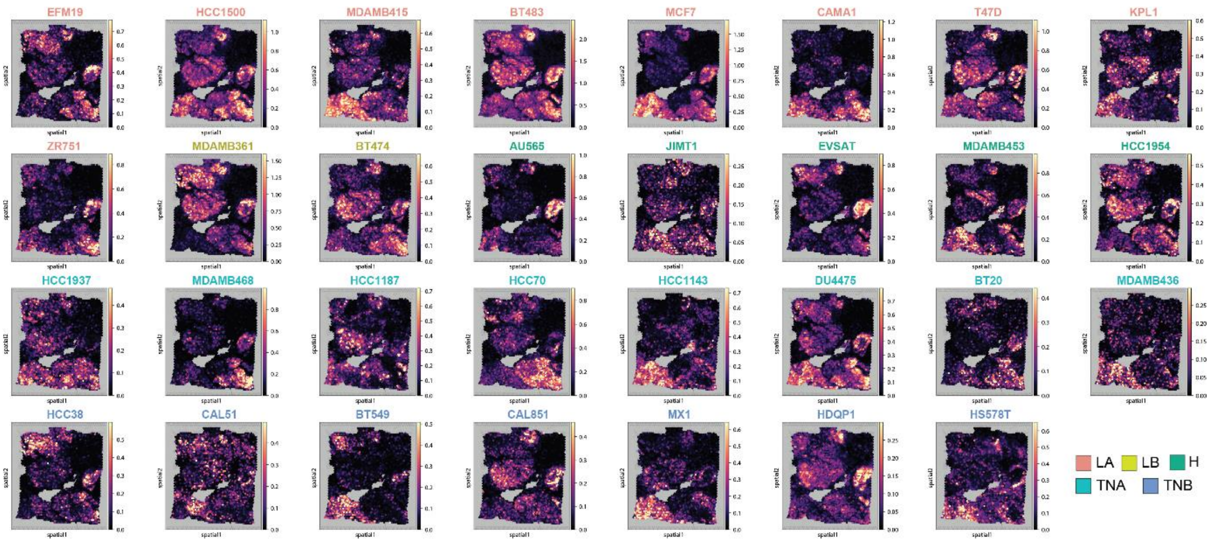

Supplementary Figure 12 - Cell line compostion from spatial transcriptomic data of the lobular BC patient shown in Supplementary Figure 10B using the Cell2Location algorithm to deconvolve each spatial tile to multiple cell lines. Cell2Location cell-line is applied to the transcriptomic data of each spatial tile to estimate its cell line composition using the BC single cell atlas. The relative abundance of each of the 31 BC cell lines estimated from Cell2Location across the tissue biopsie is represented on a colour scale with brighter spots reporting highest abundance. for the lobular BC patient for which spatial transcriptomic data was available from 10x Genomics repository. The name of each cell-line is coloured according to its BC type.

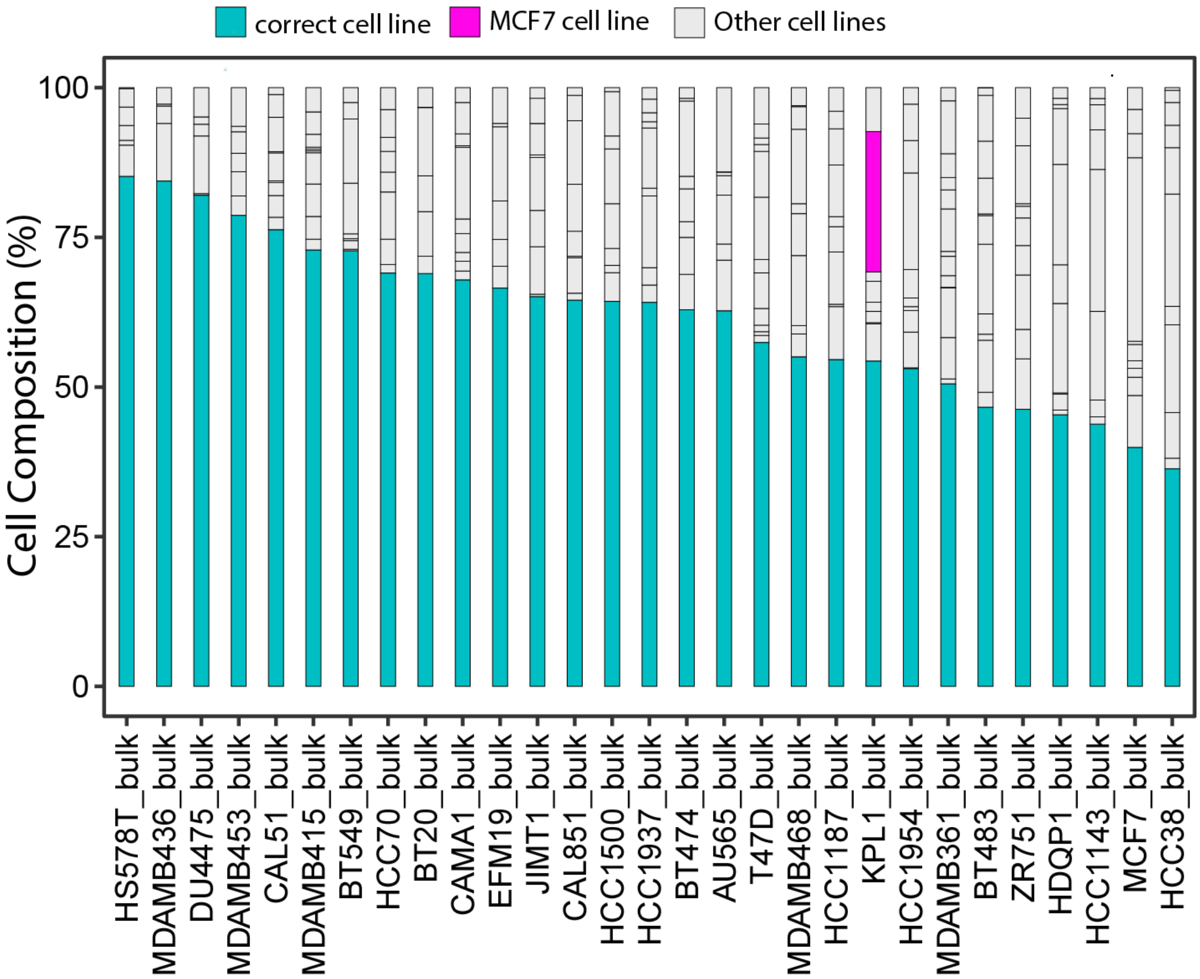

Supplementary Figure 13 – Performance in predicting cell line composition from bulk RNA-seq using the Bisque algorithm. The Bisque deconvolution algorithm was first trained on the BC single-cell atlas and then its performance estimated by predicting cell-line composition from the bulk RNA-seq of each of the 29 (out of 32) BC cell lines for which bulk data were available in the Cancer Cell Line Encyclopaedia (CCLE). The stacked bar plot reports the cell line composition predicted by Bisque from the bulk RNA-seq of each indicated cell line. Gray colour in the same stacked bar correspond to distinct cell lines with cyan corresponding to the indicated cell line on the x-axis. Interestingly, Bisque predicted that the KPL1 cell line contains a large fraction of MCF7 cell-line (purple). Indeed, the KPL1 cell line is known to be cross-contaminated with MCF7 cells in the original biobank (PMID:18304946 and PMID:20143388). Source data are provided in a Source data file.

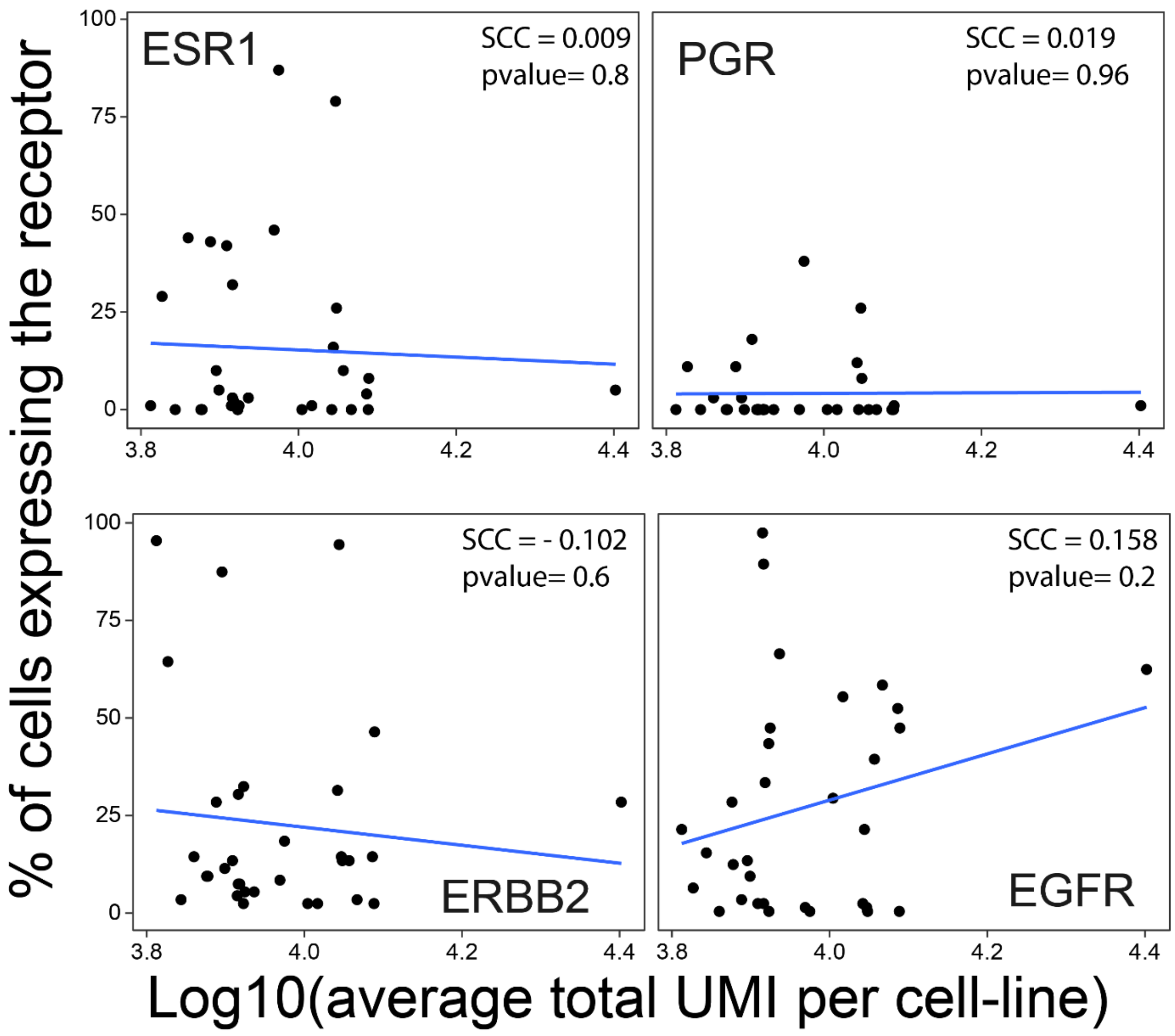

Supplementary Figure 14 –BC receptor heterogeneity in each cell-line is not correlated to sequencing depth. Spearman correlation coefficient (SCC) between percentage of cells across cell-lines expressing the indicated breast cancer receptor and average sequencing depth. For each cell-line, the percentage of cell-expressing the breast cancer receptor was estimated as the number of cells expressing the receptor (UMI>0) over the total number of sequenced cells, while average cell depth was estimated as the average number of UMI of cells in the cell-line.

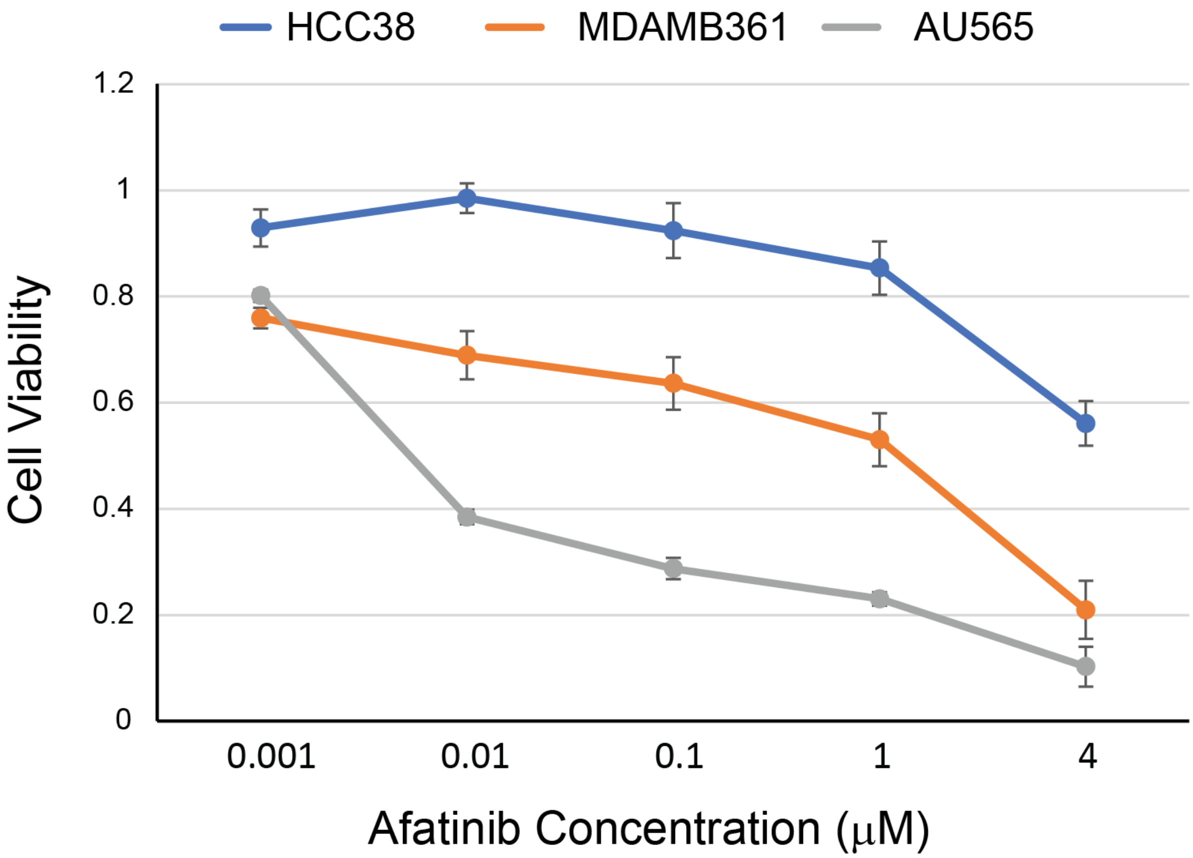

Supplementary Figure 15 – Sensitivity of the BC cell-lines to HER2 inhibitors was highly correlated with the percentage of cells in the cell line expressing *ERBB2*. Cell viability as a function of afatinib treatment for cell-lines expressing ERBB2 receptor in different proportion (i.e. high AU565, middle MDAMB361, low HCC38). Experiment was performed in triplicate for each concentration and viability was measured after 72h of treatment. Source data are provided in a Source data file.

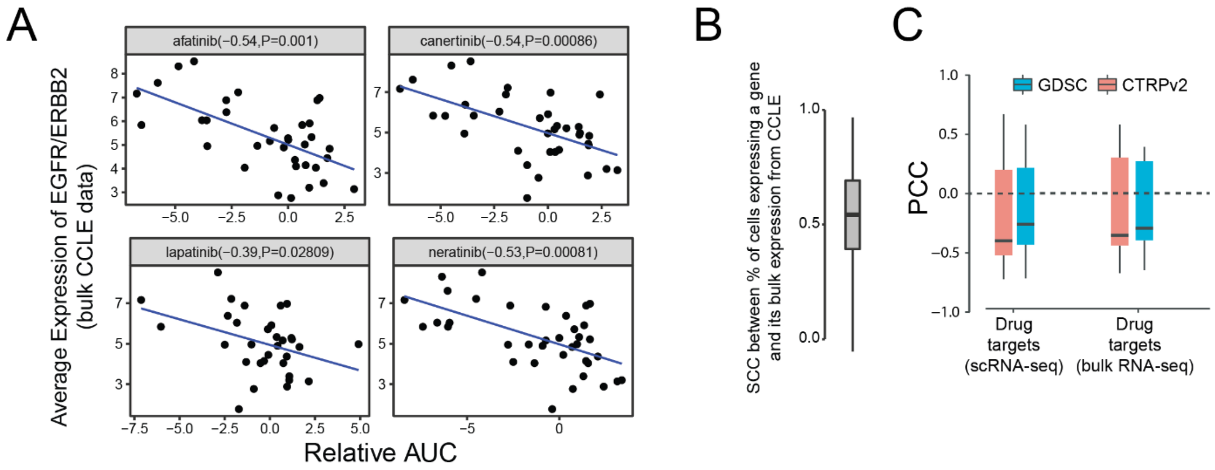

Supplementary Figure 16 – Correlation between bulk expression, single-cell expression and drug potency. (**A**) Relationship between gene expression and drug potency for four anti-HER2 drugs. Each dot corresponds to a BC cell line reporting the percentage of cells expressing ERBB2 or EGFR in the cell line [y-axis] and the experimental drug potency from the CTRPv2 database2 measured as the Aurea Under the Curve (AUC) of the dose response curve [x-axis]. Bulk expression was retrieved from CCLE. PCC (Pearson correlation coefficient) and p-value are also shown. (**B**) Spearman Correlation Coefficient (SCC) distribution between bulk gene expression and single-cell gene expression measured as percentage of gene expressing cells. Bulk gene expression was retrieved from CCLE, single cell expression from the atlas. Correlation coefficient was estimated across 29 cell-lines for which both single cell and bulk data were available. In the boxplot, n=9,756. (**C**) Box-plots reporting the distribution of PCCs between the cognate drug target expression and the potency of the drug (AUC from the CTRPv2 database, or IC50 from the GDSC dataset) across BC cell lines for 66 drugs. The cognate drug target expression was measured either as the percentage of cells in the cell line expressing it (scRNA-seq), or from the bulk gene expression profile of the cell line (bulk RNA-seq). Boxplots containing PCC distribution between a random gene and drug n=1000, while n=66 for boxplot containing PCC distribution between a drug and its cognate target gene.

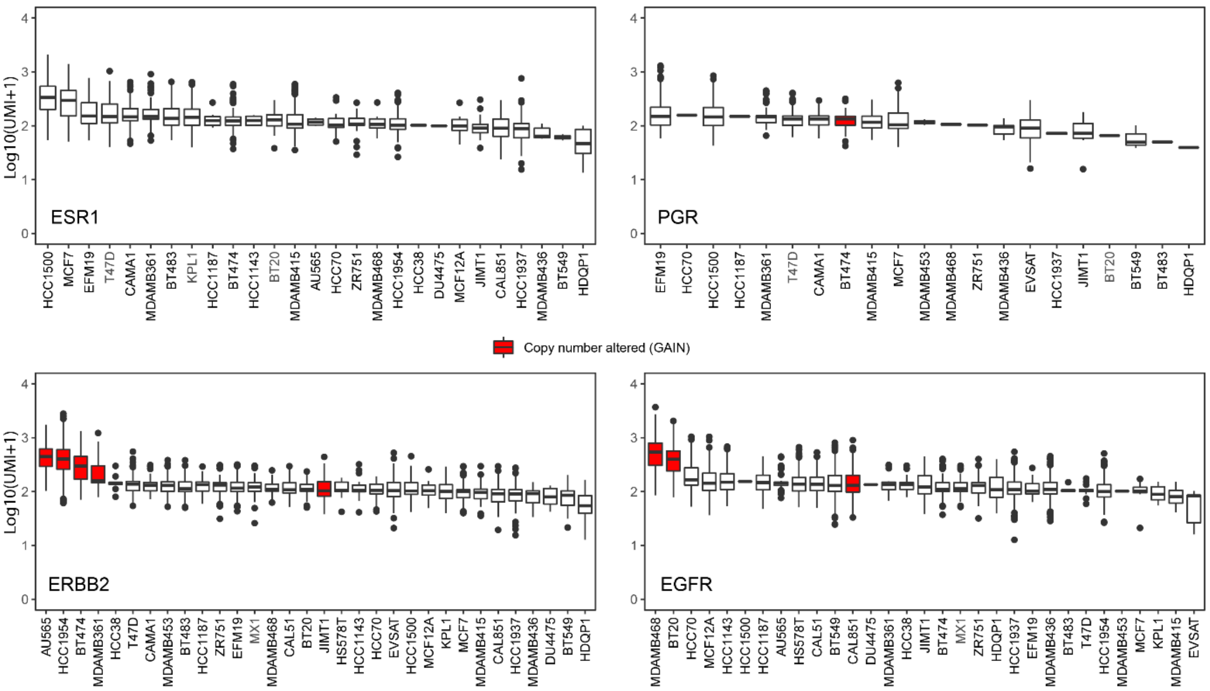

Supplementary Figure 17 – BC receptor expression level is substantially the same across cells expressing it. For each cell-line, the average expression of the indicated biomarker was estimated only among the cells expressing it. In red we highlighted those cell-lines with a copy number gain of the biomarker as reported in the COSMIC database.

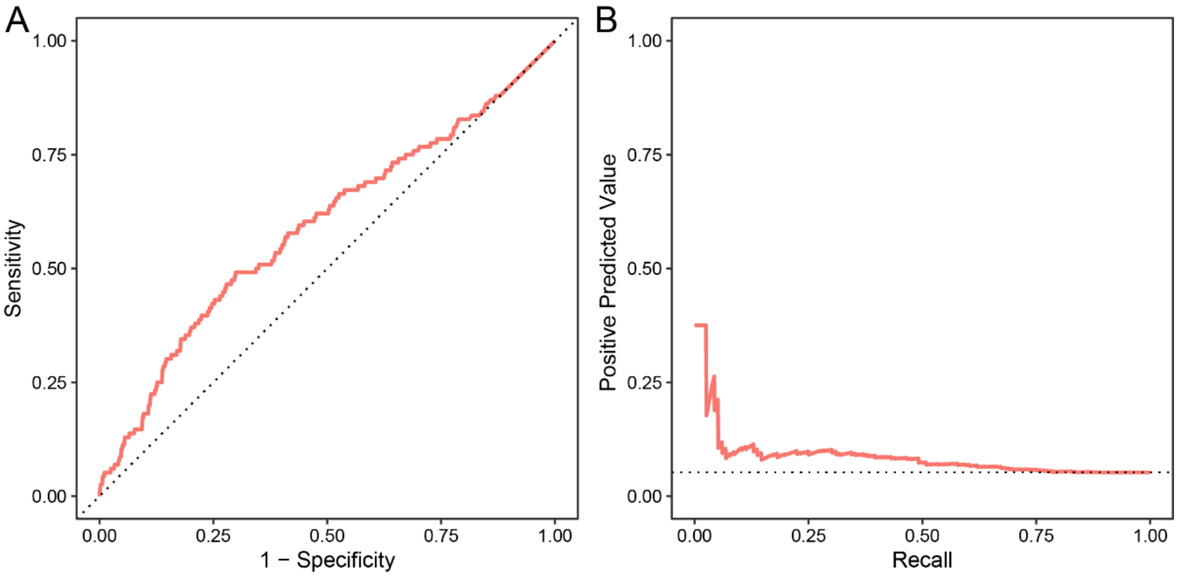

Supplementary Figure 18 – **DREEP validation using GDSC golden standard**. Performance of DREEP in predicting drug sensitivity of 32 cell lines in the atlas to 86 drugs whose drug potency was estimated in the GDSC study in terms of IC50. **(A)** ROC-curve ; **(B)** PPV (Positive Predicted Value) – Recall curve. Dashed line represents the performance of a random algorithm.

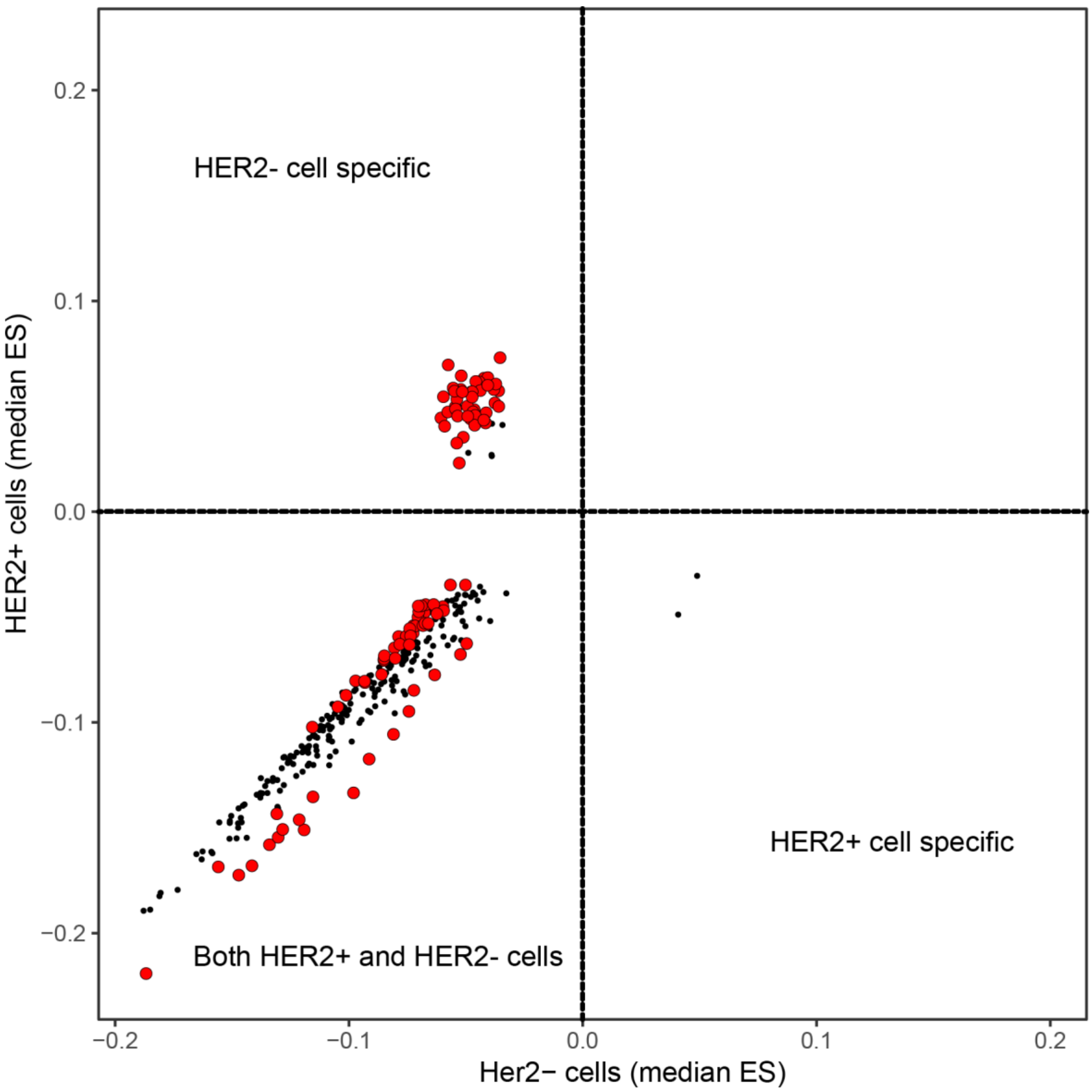

Supplementary Figure 19 – Drug sensitivity prediction using DREEP on HER2+ and HER2- cells in the MDAMB361 cell-line. Each point represents one drug whose coordinates are the median enrichment score predicted by DREEP for the drug in ERBB2 deficient cells (HER2-) [*x-axis*] and in ERBB2 expressing cells (HER2+) [*y-axis*]. The more negative the value of the enrichment score, the more potent is the predicted drug effect. The drugs with a significant enrichment score in at least one of the two subpopulations (FDR<0.01) are indicated in red. Source data are provided in a Source data file.

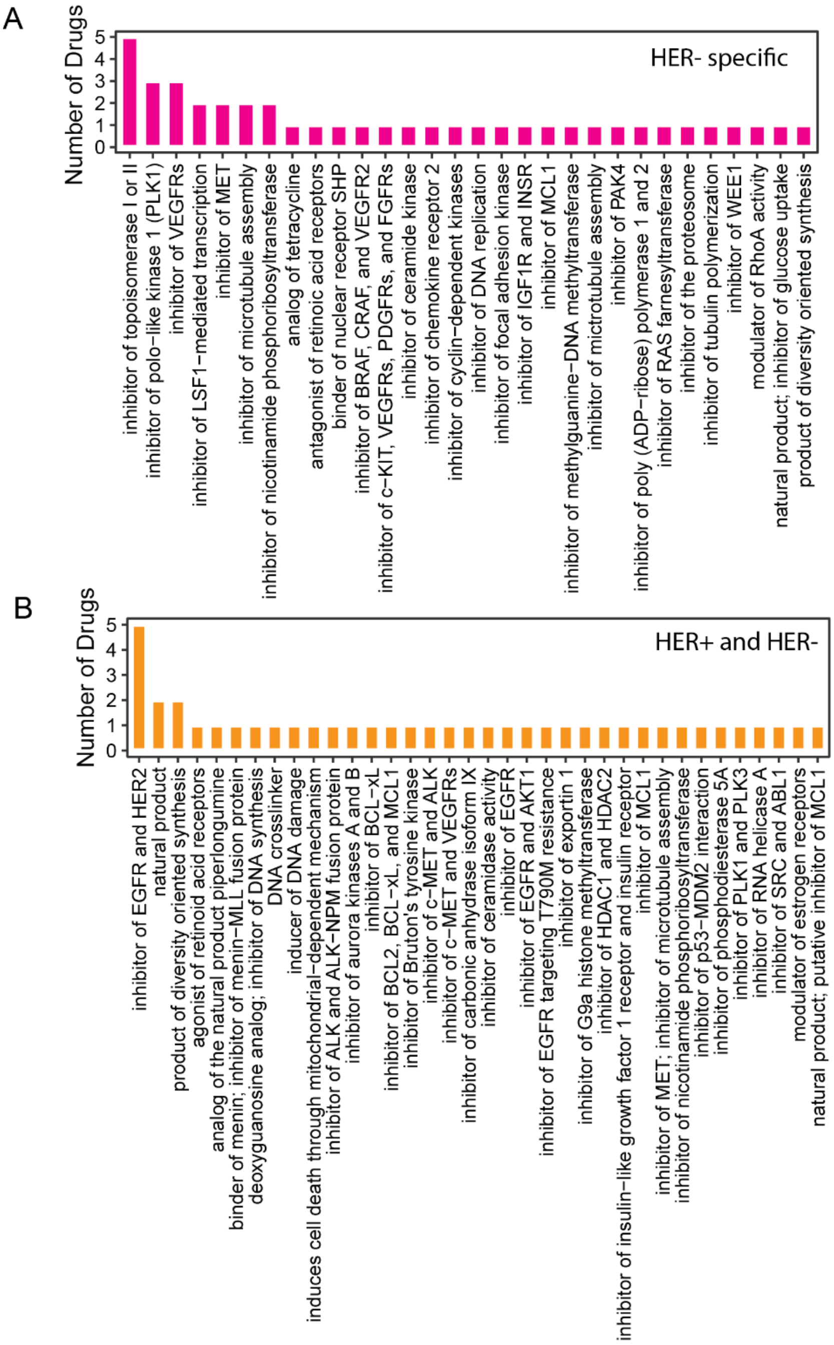

Supplementary Figure 20 – Classification of drugs predicted by the DREEP algorithm to specifically inhibit growth of the HER2- subpopulation or both HER2+ and HER2- cell populations in MDAMB361 cells. (**A**) Drugs predicted to significantly inhibit the growth of HER2- cells in the MDAMB361 cells were grouped according to their cognate targets. (**B**) Drugs predicted to significantly inhibit the growth of both HER2- and HER2+ cells in the MDAMB361 cells were grouped according to their cognate targets.

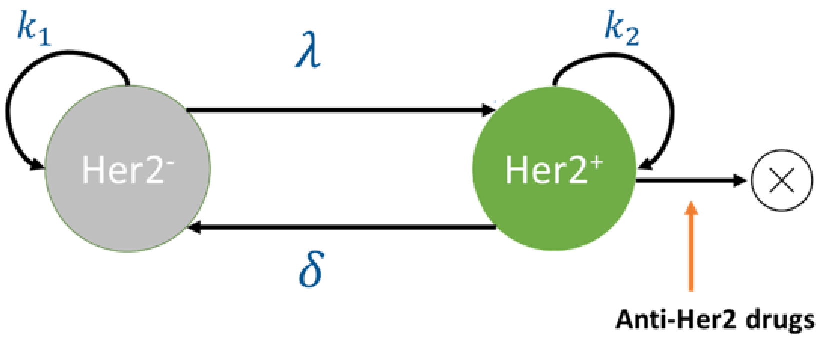

λ	0.6 $days^{-1}$
δ	0.4 $days^{-1}$
k_1	0.2 $days^{-1}$
k_2	0.2 $days^{-1}$

Supplementary Figure 21 – A two state model of interconversion of MDAMB361 cells. A two-state model of interconversion of MDAMB361 cells with arrows indicating reactions occurring at the rates reported on the arrow with values in the table.

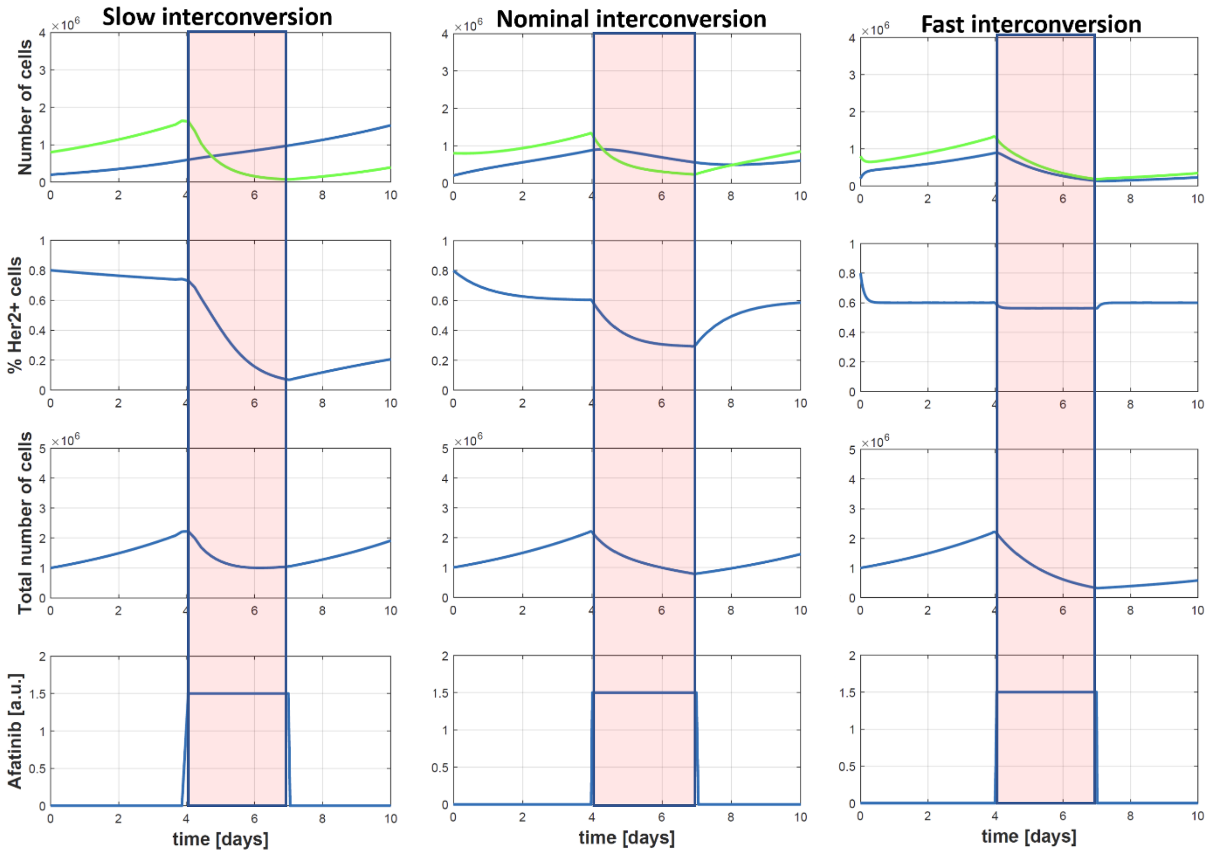

Supplementary Figure 22 – Numerical simulations of the effect of Afatinib on MDAMB361 cell line. Three different sets of parameters' values were used to investigate the effect of changing the interconversion rate on the response of MDMA261 cells to afatinib treatment. Simulations start with a total of 1 million cells, of which 0.8 million Her2+ and 0.2 million Her2-. In the first row, the green line stands for Her2+ cells and the blue line for Her2- cell.

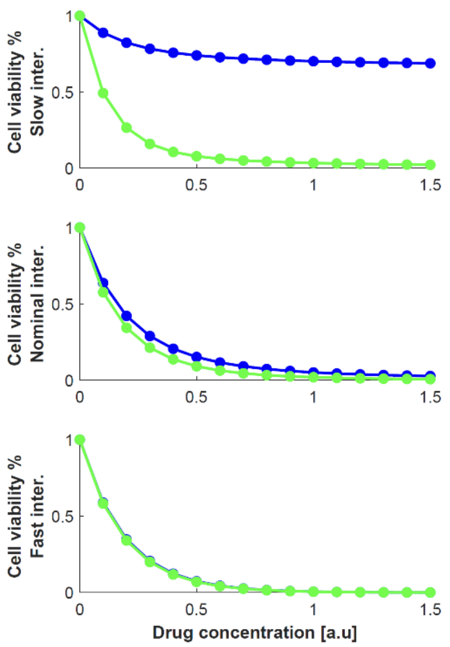

Supplementary Figure 23 – Simulated dose response curve to afatinib for MDAMB361 cells. The simulated cell viability was obtained by setting both the HER2- and HER2+ populations at $5x10^5$ cells at simulation time zero and then running the simulation with or without Afatinib at the indicated concentrations for 10 days, and then dividing the resulting number of Her2- cells (resp. Her2+) treated with Afatinib by the number of Her2- cells (resp. Her2+) grown in the absence of Afatinib. Cell viability was simulated for the three different set of parameters' values as in Suppl. Fig. 14. Green line refers to Her2+ cells while the blue line to Her2-cells.

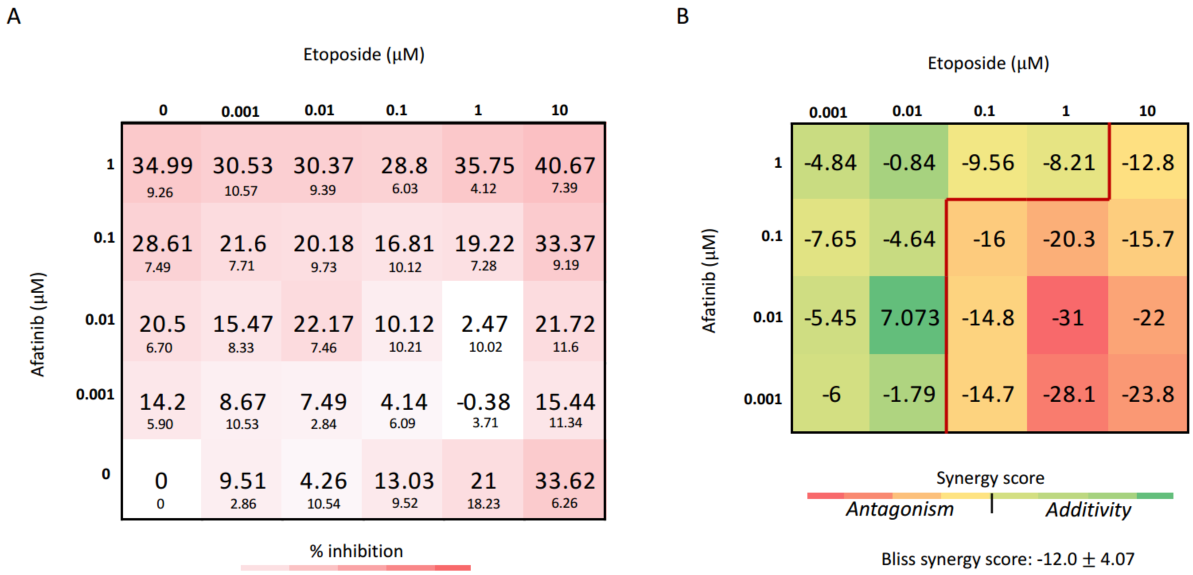

Supplementary Figure 24 - Drug combination assays with the estimated Bliss synergy scores. (**A**) Grid plot showing the percentage of inhibition (DMSO normalized) of the MDA-MB-361 cells upon 72 hr of either Afatinib or Etoposide treatment as single agent, and in all possible dose combinations (S.D. is the smaller number). (**B**) Grid plot with the Bliss synergy score estimation at each Afatinib and Etoposide dose pair. Synergy scores at each dose pair and the overall Bliss synergy score (with 95% CI) were computed by SynergyFinder version 2.0.

Supplementary Table 01 – Characteristics of the breast cancer cell-lines sequenced in this study. (A) Official name of the cell line; (B) ER status; (C) PR status; (D) HER2 status (E) BRCA1 mutational status; (F) BC

CCLs	ER	PR	HER2	BRCA1	Subtype	Growth.medium	COSMIC ID	derivation_	site	# cell
AU565	-	-	+	WT	H	RPMI 2mM L-Glutamine, 10 %FBS	COSM910704	metastatic site: malignant pleural effusion	ATCC	596
BT20	-	-	-	WT	TNA	MEM 2mM L-Glutamine, 10 %FBS	COSM906801	mammary gland/breast	ATCC	638
BT474	+	+	+	WT	LB	DMEM 2mM L-Glutamine, 10 %FBS	COSM946359	mammary gland; breast/duct	ATCC	2158
BT483	+	+/-	-	WT	LA	RPMI 2mM L-Glutamine, 0.01 mg/mL bovine insulin, 20 %FBS	COSM949093	mammary gland; breast	ATCC	385
BT549	-	-	-	WT	TNB	RPMI 2mM L-Glutamine, 10 %FBS	COSM905951	mammary gland; breast	ATCC	2296
CAL51	-	-	-	WT	TNB	DMEM 2mM L-Glutamine, 10 %FBS	COSM910927	metastatic site: pleural effusion	DSMZ	1260
CAL851	-	-	-	WT	TNB	DMEM 2mM L-Glutamine, 10 %FBS, 1mM sodium pyruvate	COSM910852	relapsing invasive galactophoric breast adenocarcinoma	DSMZ	995
CAMA1	+	+/-	-	WT	LA	MEM 2mM L-Glutamine, 10 %FBS, 1% NEAA	COSM946382	metastatic site: pleural effusion	ATCC	823
DU4475	-	-	-	WT	TNA	RPMI 2mM L-Glutamine, 20 %FBS	COSM906844	metastatic site: skin carcinoma	ATCC	415
EFM19	+	+	-	ND	LA	RPMI 2mM L-Glutamine, 10 %FBS	COSM906851	metastatic site: pleural effusion	DSMZ	1316
EVSAT	-	+/-	+	ND	H	MEM 2mM L-Glutamine, 10 %FBS, 25 mM HEPES	NA	metastatic site: malignant ascitic effusion	DSMZ	441
HCC1143	-	-	-	ND	TNA	RPMI 2mM L-Glutamine, 20 %FBS	COSM749710	mammary gland; breast/duct	ATCC	1280
HCC1187	-	-	-	ND	TNA	RPMI 2mM L-Glutamine, 10 %FBS	COSM749711	mammary gland; breast	ATCC	1147
HCC1500	+	+/-	-	ND	LA	RPMI 2mM L-Glutamine, 10 %FBS	NA	mammary gland; breast/duct	ATCC	615
HCC1937	-	-	-	MU	TNA	RPMI 2mM L-Glutamine, 10 %FBS	COSM749714	mammary gland; breast/duct	ATCC	1028
HCC1954	-	-	+	WT	H	RPMI 2mM L-Glutamine, 10 %FBS	COSM749709	mammary gland; breast/duct	ATCC	1623
HCC38	-	-	-	ND	TNB	RPMI 2mM L-Glutamine, 10 %FBS	COSM749717	mammary gland; breast/duct	ATCC	629
HCC70	-	-	-	WT	TNA	RPMI 2mM L-Glutamine, 10 %FBS	COSM907048	mammary gland; breast/duct	ATCC	2818

HDQP1	-	-	-	MU	TNB	DMEM 2mM L-Glutamine, 10 %FBS	NA	primary ductal infiltrating breast carcinoma	DSMZ	110
HS578T	-	-	-	WT	TNB	RPMI 2mM L-Glutamine, 10 %FBS	COSM905957	mammary gland/breast	ATCC	879
JIMT1	-	-	+	ND	H	DMEM 2mM L-Glutamine, 10 %FBS	NA	metastatic site: pleural effusion	DSMZ	568
KPL1	+	-	-	ND	LA	DMEM 2mM L-Glutamine, 10 %FBS	NA	metastatic site: pleural fluid	DSMZ	942
MCF12A	-	-	-	ND	Basal-like	DMEM/F12 - 1:1 mixture of Dulbecco's Modified Eagle Medium and Nutrient Mixture F-12, 20 ng/mL EGF, 0.03 µM ITS, 10% FBS	NA	mammary gland; non-tumorigenic, fibrocystic breast disease	ATCC	1744
MCF7	+	+	-	WT	LA	MEM 2mM L-Glutamine, 10 %FBS, 1% NEAA	COSM905946	metastatic site: pleural effusion	ATCC	839
MDAMB361	+	+/-	+	WT	LB	RPMI 2mM L-Glutamine, 20 %FBS	COSM908121	metastatic site: brain	ATCC	753
MDAMB415	+	+/-	-	WT	LA	RPMI 2mM L-Glutamine, 10 %FBS, 10 µg/mL insulin, 10 µg/mL glutathione, 15% FBS	COSM924240	metastatic site: pleural effusion	ATCC	877
MDAMB436	-	-	-	MU	TNA	RPMI 2mM L-Glutamine, 10 %FBS	COSM1240172	metastatic site: pleural effusion	ATCC	2821
MDAMB453	-	-	+	WT	H	RPMI 2mM L-Glutamine, 10 %FBS	COSM908122	metastatic site: pericardial effusion	ATCC	1119
MDAMB468	-	-	-	WT	TNA	RPMI 2mM L-Glutamine, 10 %FBS	COSM908123	metastatic site: pleural effusion	ATCC	1573
MX1	-	-	-	WT	TNB	DMEM/F12 - 1:1 mixture of Dulbecco's Modified Eagle Medium and Nutrient Mixture F-12, 2 mM L-Glutamine, 10% FBS	NA	originated from primary breast cancer. Primary xenotransplan t from an infiltrating duct carcinoma	NIH-DCTD	820
T47D	+	+	-	WT	LA	RPMI 2mM L-Glutamine, 10 %FBS, 1 mM sodium pyruvate, 10 mM HEPES	COSM905945	metastatic site: pleural effusion	ATCC	825
ZR751	+	+/-	-	WT	LA	RPMI 2mM L-Glutamine, 10 %FBS	NA	metastatic site: ascites	ATCC	943

Supplementary Table 02 – STR profiling by means of the AmpFISTR Identifier Plus PCR Amplification kit (Applied Biosystems). (A) official name of the cell line; (B) ER status; (C)-(T) markers analyzed with the AmpFISTR Identifier Plus PCR Amplification kit; (U) markers used for cell line profiling and (V) the similarity score obtained.

CELL LINE	External Reference	AMEL	CSF1PO	D13S317	D16S539	D18S51	D19S433	D21S11	D2S1338	D3S1358	D5S818	D7S820	D8S1179	FGA	TH01	TPOX	VWA	PENTAD	PENTAE	Loci used for comparison	Similarity Score
AU-565		20 20	12 12	11 12	9 9	10 13	14 14	30 30.2	20 25	17 17	9 12	9 12	12 12	20 20	8 9	8 11	17 17				
	GNE_1	20 20	12 12	11 11	9 9	10 13		30 30.2		17 17	9 12	9 12	12 12	20 20	8 9	8 11	17 17	9 12	10 11	14	96.4
	GNE_2	20 20	12 12	11 12	9 9	10 13		30 30.2		17 17	9 12	9 12	11 12	20 20	8 9	8 11	17 17	9 12	10 11	14	96.4
BT-20		20 20	12 12	11 11	11 14	17 17	15 15	28 29	19 19	17 17	12 12	10 10	12 12	22 24	7 9.3	11 11	16 17				
	ATCC_DB	20 20	12 12	11 11	11 14						12 12	10 10			7 9.3	11 11	16 17			9	100
	GNE	20 20	12 12	11 11	11 11	17 17		28 29		17 17	12 12	10 10	12 12	22 24	7 9.3	11 11	16 17	10 11	11 13	14	96.4
BT-474		20 20	10 11	11 11	9 11	13 18	14 14.2	28 32.2	19 19	17 17	11 13	9 12	10 12	22 25	7 7	8 8	15 16				
	GNE	20 20	10 11	11 11	9 11	13 18	14 17	28 32.2	19 19	17 17	11 13	9 12	10 12	22 25	7 7	8 8	15 16	9 14	5 5	16	96.9
BT-483		20 20	9 12	8 12	11 12	13 15	14 15	30 30.2	17 23	14 17	11 11	9 10	15 15	23 26	5 9.3	11 11	17 17				
	ATCC_db	20 20	9 12	8 12	11 12						11 11	9 10			5 9.3	11 11	17 17			9	100
	GNE	20 20	9 12	8 12	11 12	13 15		30 30.2		14 17	11 11	9 10	15 15	23 26	5 9.3	8 11	17 17	9 11	14 20	14	96.4
BT-549		20 20	10 12	11 11	8 8	15 15	15.2 15.2	32.2 32.2	17 17	18 18	11 11	9 10	14 16	19 19	9.3 9.3	8 8	15 15				
	CLS_DB	20 20	10 12	11 11	8 8	15 15	15.2 15.2	32.2 32.2	17 17	18 18	11 11	9 10	14 16	19 19	9.3 9.3	8 8	15 15			16	100
	GNE	20 20	10 12	11 11	8 8	15 15		32.2 32.2		18 18	11 11	9 10	14 16	19 19	9.3 9.3	8 8	15 15	13 13	14	14	100
CAL-51		20 20	11 12	11 13	11 13	15 16 17	12 14	31 32	23 24	16 17	12 13	7 12	14 14	22 23	7 7	8 9	18 18				
	GNE	20 20	11 12	11 13	11 13	15 17		31 32		16 17	12 13	7 12	14 14	22 23	7 7	8 9	18 18	10 14	7 12	14	98.2
CAL-85-1		20 20	12 12	11 11	11 13	14 14	14.2 15	30.2 32.2	20 20	16 16	11 11	9 10	15 15	24 24	6 6	8 11	18 18				
	GNE	20 20	12 12	11 11	11 13	14 14		30.2 32.2		16 16	11 11	9 10	15 15	24 24	6 6	8 11	18 18	9 9	11 11	14	100
CAMA-1		20 20	10 12	12 12	11 11	14 15	13 13	32.2 32.2	17 21	18 18	12 13	8 11	12 13	19 25	8 9.3	8 8	15 15				
	GNE	20 20	10 12	12 12	11 11	14 15		32.2 32.2		18 18	12 13	8 11	12 13	19 25	8 9.3	8 8	15 15	10 11	12 14	14	100
DU-4475		20 20	9 12	11 14	11 12	14 16	14 14	29 31.2	20 25	14 16	11 11	9 10	10 13	22 25	6 8	8 8	17 17				
	GNE	20 20	9 12	11 14	11 12	14 16		29 31.2		14 16	11 11	9 10	10 13	22 25	6 8	8 8	17 17	13 14	7 13	14	100
EFM-19		20 20	9 9	8 12	11 12	13 13	12 14	28 28	19 20	18 18	11 11	9 9	13 14	22 22	7 8	8 12	14 14				
	GNE	20 20	9 9	8 12	11 12	13 13		28 28		18 18	11 11	9 9	13 14	22 22	7 8	8 12	14 14	9 13	13 14	14	100
EVSA-T		20 20	11 12	11 12	9 9	12 17	13 13	30 31.2	24 24	14 17	12 13	10 12	16 16	21 24 25	6 6	8 8	15 16				
	DSMZ_DB	20 20	11 12	11 12	9 9						12 13	10 12			6 6	8 8	15 16			9	100
	GNE	20 20	11 12	12 12	9 9	12 17		30 31.2		14 17	12 13	10 12	16 16	21 24 25	6 6	8 8	15 16	12 14	12 16	14	96.6
HCC-1143		20 20	10 10	12 12	11 13	14 17	13 16.2	30.2 30.2	20 23	16 16	11 11	12 12	13 13	21 21	9.3 9.3	12 12	16 16				
	GNE	20 20	10 10	12 12	11 13	14 14		30.2 30.2		16 16	11 11	12 12	13 13	21 21	9.3 9.3	12 12	16 16	8 8	10 10	14	96.4
HCC1187		20 20	13 13	11 11	10 10	17 17		29 30		15 15	12 12	8 11	11 11	23 23	6 6	8 8	19 19	11 11	16 16		
	GNE	20 20	13 13	11 11	10 10	17 17		29 30		15 15	12 12	8 11	11 11	23 23	6 6	8 8	19 19	11 11	16 16	16	100
HCC1500		20 20	10 10	10 10	9 10	13 16	13 13	27 30	18 23	15 15	11 13	11 11	13 14	23 23	9 9	8 8	13 16				
	GNE	20 20	10 10	10 10	9 10	13 13		27 30		15 15	11 13	11 11	13 14	23 23	9 9	8 8	13 16	2.2 5	7 7	14	96.4
HCC1937		20 20	12 12	13 13	13 14	12 12	14 15	28 28	25 25	18 18	12 12	9 10	12 13	20 22	6 6	11 11	16 17				
	GNE	20 20	12 12	13 13	13 14	12 12		28 28		18 18	12 12	9 10	12 13	20 22	6 6	11 11	16 17	9 9	13 13	14	100
HCC1954		20 20	10 10	8 9	9 11	14 18		28 32.2		15 16	11 11	10 11	12 15	22 23	6 7	8 9	18 19	9 12	12 16		
	GNE	20 20	10 10	8 9	9 11	14 18		28 32.2		15 16	11 11	10 11	12 15	22 23	6 7	8 9	18 19	9 12	12 16	16	100
HCC38		20 20	12 12	12 14	10 14	15 15	13 15	27 28	17 19	18 18	9 9	10 10	8 10	24 24	9.3 9.3	9 12	16 17				
	GNE	20 20	12 12	12 14	10 14	15 15		27 28		18 18	9 9	10 10	8 10	24 24	9.3 9.3	9 12	16 17	9 9	5 11	14	100
HCC70		20 20	10 14	12 12	9 13	13 16		30 30		16 17	12 13	10 11	13 13	19.2 24	9 9	10 10	13 15	10 10	9 16		
	ATCC_DB	20 20	10 14	12 12	9 13						12 13	10 11			9 9	10 10	13 15			9	100
	GNE	20 20	10 14	12 12	13 13	13 16		30 30		16 17	13 13	10 11	13 13	19.2 24	9 9	9 10	13 15	10 10	9 16	16	90.6
HDQ-P1		20 20	11 11	12 12	11 11	13 13	13 13	29 29	17 17	15 15	11 11	11 12	12 15	22 22	9.3 9.3	8 8	16 18				
	GNE	20 20	11 11	12 12	11 11	13 13		29 29		15 15	11 11	11 12	15 15	22 22	9.3 9.3	8 8	16 18	11 11	11 13	14	96.4
Hs-578-T		20 20	13 13	11 11	9 12	16 16	14 15	29 32.2	17 26	16 17	11 11	10 10	13 13	23 24	9 9.3	8 8	17 17				
	GNE	20 20	13 13	11 11	9 12	16 16		29 32.2		16 17	11 11	10 10	13 13	23 24	9 9.3	8 8	17 17	8 13	13 14	14	100
	Lorenzi_paper	20 20	13 13	11 11	9 12	16 16	14 15	29 32.2	17 26	16 17	11 11	10 10	13 13	23 24	9 9.3	8 8	17 17			16	100

JIMT-1		20 20	12 13	8 13	11 11	10 10	12 13	27 31.2	24 24	18 18	12 13 14	12 12	10 13	21 23	7 7	8 8	18 18				
	GNE	20 20	12 13	8 13	11 11	10 10		27 31.2		18 18	12 13 14	12 12	10 13	21 23	7 7	8 8	18 18	9 12	7 19	14	100
KPL-1		20 20	10 10	11 11	11 12	14 14	13 14	30 30	21 23	16 16	11 12	8 9	10 14	23 25	6 6	9 12	14 15				
	DSMZ_DB	20 20	10 10	10 11	11 12						11 12	8 9			6 6	9 12	14 15			9	94.4
MCF-12A		20 20	10 11	9 11	9 12	18 18	13 15	28 30	19 19	16 18	11 13	8 11	10 13	23 26	7 7	8 8	18 18				
	DSMZ_DB	20 20	10 11	9 11	9 12						11 13	8 11			7 7	8 8	18 18			9	100
MCF7		20 20	10 10	11 11	11 12	14 14	13 14	30 30	21 23	16 16	11 12	8 9	10 14	23 25	6 6	9 12	14 15				
	GNE	20 20	10 10	11 11	11 12	14 14		30 30		16 16	11 12	8 9	10 14	23 25	6 6	9 12	14 15	12 12	7 12	14	100
	Lorenzi_paper	20 20	10 10	11 11	11 12	14 14	13 14	30 30	21 23	16 16	11 12	8 9	10 14	23 25	6 6	9 12	14 15			16	100
MDA-MB-361		20 20	12 12	11 11	11 12	12 15	12.2 14	30 32.2	19 20	16 16	10 11	9 12	15 15	20 24	9.3 9.3	8 11	17 17				
	GNE	20 20	12 12	11 11	11 12	12 15		30 32.2		16 16	10 11	9 12	15 15	20 24	9.3 9.3	8 11	17 17	12 13	8 14	14	100
MDA-MB-415		20 20	10 12	11 13	12 13	13 13		27 28		17 17	11 13	9 10	8 12	22 22	7 9.3	8 8	17 17	9 10	7 16		
	GNE	20 20	10 12	11 13	13 13	13 13		27 28		17 17	11 13	9 10	8 12	22 22	7 9.3	8 8	17 17	9 10	7 16	16	96.9
MDA-MB-436		20 20	12 12	10 10	9 11	12 12	13 13	30 31.2	23 23	18 18	13 13	10 10	10 14	24 24	9.3 9.3	8 8	14 20				
	ATCC_DB	20 20	12 12	10 10	9 11						13 13	10 10			9.3 9.3	8 8	14 20			9	100
	GNE	20 20	12 12	10 10	9 9	12 12		30 31.2		18 18	13 13	10 10	10 14	24 24	9.3 9.3	8 8	14 20	9 9	10 12	14	96.4
MDA-MB-453		20 20	10 12	12 12	9 9	15 20	13 14	29 31	23 24	15 15	11 11	10 10	10 12	18 23	6 6	10 10	17 18				
	GNE	20 20	10 12	12 12	9 9	15 20		29 31		15 15	11 11	10 10	10 12	18 23	6 6	10 10	17 18	9 10	11 11	14	100
MDA-MB-468		20 20	12 12	12 12	9 9	17 17		27 28		15 15	12 12	8 8	13 13	23 23	7 7	8 9 10	18 18	8 10	5 5		
	GNE	20 20	12 12	12 12	9 9	17 17		27 28		15 15	12 12	8 8	13 13	23 23	7 7	8 9	18 18	8 10	5 5	16	98.5
MX-1		20 20	11 11	11 11	12 12	12 16		29 32		15 15	12 12	11 11	11 11	20 20	7 9	8 8	17 17	9 11	14 14		
	GNE	20 20	11 11	11 11	12 12	12 16		29 32		15 15	12 12	11 11	11 11	20 20	7 9	8 8	17 17	6 9 11	14 14	16	98.5
T47D		20 20	11 13	12 12	10 10	17 17	14 14	28 31	24 24	15 17	12 12	11 11	13 13	23 23	6 6	11 11	14 14				
	GNE	20 20	11 13	12 12	10 10	17 17		28 31		15 17	12 12	11 11	13 13	23 23	6 6	11 11	14 14	10 12	7 14	14	100
	Lorenzi_paper	20 20	11 13	12 12	10 10	17 17	14 14	28 31	24 24	15 17	12 12	11 11	13 13	23 23	6 6	11 11	14 14			16	100

Supplementary Table 03 – Gene Set Enrichment Analysis (GSEA) for the basal gene signature across sequenced cell-lines. (A) Official name of the cell line; (B) Breast cancer subtype; (C) p-value of the Enirchment Score as reported by GSEA (D) False Discovery Rate (Benjamini-Hochberg); (E) Enrichement score reported by GSEA.

**ccl**	**type**	**pval**	**FDR**	**ES**
MCF12A	Basal-like	1E-10	3.2E-09	0.779692
HCC70	TNA	2.76E-09	4.41E-08	0.757287
HCC1500	LA	2.67E-08	2.84E-07	-0.70047
MDAMB46	TNA	5.38E-06	4.31E-05	0.644507
EVSAT	H	0.000101	0.000645	-0.58903
MDAMB45	H	0.000173	0.000925	-0.59646
CAL851	TNB	0.000583	0.002666	0.594522
BT549	TNB	0.000938	0.003751	-0.5503
HCC1143	TNA	0.001419	0.005044	0.55508
HCC1937	TNA	0.003336	0.010676	0.550628
MDAMB36	LB	0.006185	0.017992	-0.51439
BT483	LA	0.009575	0.025532	-0.50704
CAMA1	LA	0.011446	0.025907	-0.50594
MCF7	LA	0.010899	0.025907	-0.48215
MX1	TNB	0.012144	0.025907	0.465368
BT20	TNA	0.022318	0.044636	0.459129
HCC1187	TNA	0.024195	0.045543	0.43431
T47D	LA	0.03517	0.062525	-0.43532
HCC1954	H	0.037147	0.062564	0.467783
KPL1	LA	0.03974	0.063585	-0.43366
BT474	LB	0.049505	0.075436	-0.44929
EFM19	LA	0.100939	0.14682	-0.3772
CAL51	TNB	0.141491	0.196858	-0.38661
DU4475	TNA	0.218147	0.290862	-0.35029
ZR751	LA	0.24238	0.310247	-0.34939
MDAMB43	TNA	0.414286	0.50989	-0.2988
JIMT1	H	0.497561	0.589702	0.323361
HDQP1	TNB	0.606591	0.67931	0.319407
HS578T	TNB	0.615625	0.67931	-0.26047
MDAMB41	LA	0.90843	0.968992	0.202724
AU565	H	0.990333	1	-0.1597
HCC38	TNB	1	1	0.118846

Supplementary Table 04 – ANOVA test for each of the 22 scRNA-seq biomarker genes across 937 TCGA patients as reported in Figure 2D. (A) Official gene symbol; (B) p-value of the ANOVA test for each of the 22 cluster biomarkers across the 937 TGCA patients as reported in Figure 2D; (C) False Discovery Rate [Benjamini-Hochberg]

Official Gene Symbol	p-value	FDR
TCF7	2.72099E-19	3.74136E-19
DHRS2	1.15188E-39	3.6202E-39
CCL2	1.13187E-13	1.31059E-13
HSD17B1	1.99142E-10	2.19056E-10
SCGB2A2	3.61171E-46	1.32429E-45
H2BC11	1.10001E-36	3.02503E-36
KLK10	2.80751E-63	2.05884E-62
CLCA2	5.53541E-54	3.04447E-53
BCAS3	3.19593E-28	5.8592E-28
MAGEA4	1.20041E-34	2.40083E-34
XAGE2	6.74841E-35	1.48465E-34
SPON2	2.62598E-14	3.20953E-14
PIP	1.05237E-53	4.63042E-53
PDPN	1.65105E-08	1.72967E-08
ACTG2	1.23233E-35	3.01237E-35
COL1A2	6.82624E-22	1.00118E-21
PLCXD3	1.29093E-22	2.0286E-22
IRS2	9.69104E-17	1.25413E-16
APOD	1.06139E-24	1.79621E-24
S100A2	1.56758E-65	1.72434E-64
PSPHP1	0.000116267	0.000116267
DRAIC	2.54488E-98	5.59873E-97

Supplementary Table 05 – MX-1 and Hs578T sensitivity to cisplatin: (A) Assayed cell line name, or background well; (B) Biological replicate number; (C) Drug used for treatment or DMSO negative control; (D) Drug concentration or DMSO %; (E) Measured luminescence intensity following 72h treatment. Luminescence level is a measured of cell viability as assessed by Promega CellTiter-Glo® Luminescent Cell Viability Assay and measured by the GloMax® Discover instrument from Promega.

Sample	Replicate	Treatment	Concentration_	Luminescence_intensity
Hs578T	1	DMSO	DMSO 0.2%	200200
Hs578T	2	DMSO	DMSO 0.2%	170800
Hs578T	3	DMSO	DMSO 0.2%	188600
Hs578T	1	cisplatin	20	2920
Hs578T	2	cisplatin	20	3531
Hs578T	3	cisplatin	20	3310
Hs578T	1	cisplatin	10	6452
Hs578T	2	cisplatin	10	5481
Hs578T	3	cisplatin	10	5872
Hs578T	1	cisplatin	1	84680
Hs578T	2	cisplatin	1	87150
Hs578T	3	cisplatin	1	99280
Hs578T	1	cisplatin	0.1	102100
Hs578T	2	cisplatin	0.1	101400
Hs578T	3	cisplatin	0.1	107300
Hs578T	1	cisplatin	1	108300
Hs578T	2	cisplatin	1	103100
Hs578T	3	cisplatin	1	98860
MX-1	1	DMSO	DMSO 0.2%	288300
MX-1	2	DMSO	DMSO 0.2%	277600
MX-1	3	DMSO	DMSO 0.2%	278400
MX-1	1	cisplatin	20	135800
MX-1	2	cisplatin	20	192200
MX-1	3	cisplatin	20	176100
MX-1	1	cisplatin	10	224800
MX-1	2	cisplatin	10	218800
MX-1	3	cisplatin	10	180100
MX-1	1	cisplatin	1	267500
MX-1	2	cisplatin	1	283600
MX-1	3	cisplatin	1	276300
MX-1	1	cisplatin	0.1	263700
MX-1	2	cisplatin	0.1	267300
MX-1	3	cisplatin	0.1	273500
MX-1	1	cisplatin	1	268700
MX-1	2	cisplatin	1	248800
MX-1	3	cisplatin	1	285800
background	1	no	no	40

background	2	no	no	60
background	3	no	no	50

Supplementary Table 06 – PAM50 classification of pseudobulk of 5 TNBC patients. (A)Patient identifier; (B-D) Probability according to PAM50 of the sample to be of the Basal Breast Cancer subtype [B], HER2-enriched [C], Luminal A [D] and Luminal B [E]. in red the highest probability for each patient

Patient ID	BRCA_Basal	BRCA_Her2	BRCA_LumA	BRCA_LumB
TNBC1	0.9944	0.0005	0.0049	0.0002
TNBC2	0.9925	0.0005	0.0065	0.0005
TNBC3	0.9969	0.0004	0.0018	0.0009
TNBC4	0.9874	0.0004	0.0118	0.0003
TNBC5	0.0422	0.4750	0.2296	0.2532

Supplementary Table 07 – Expected heterogenity in the expression of the 4 Breast Cancer biomarker genes assuming a Poisson sampling of single cell sequencing data. For each cell line, we computed an empirical p-value for each of the four biomarkers, by randomly sampling from N (number of cells in the cell line) Poisson distributions using the estimated lambdas as described in the Methods. We obtained a "simulated" vector of counts, from which we computed the proportion of zero counts. This process was repeated 10,000 times to obtain an empirical distribution of the proportion of zero counts, which we then used to compute the empirical p-value. (A) Official name of the cell line; (B) ESR1 p-value; (C) PGR p-value; (D) ERBB2 p-value (E) EGFR p-value.

ccl	ESR1.pval	PGR.pval	ERBB2.pval	EGFR.pval
CAL51	0	0	0.4221	0.0004
HS578T	0	0	0.3459	0.1145
EFM19	0	0	0.3818	0.155
MX1	0	0	0.4071	0.2169
HCC1500	0	0	0.5949	0.3689
KPL1	0.0025	0	0.2875	0.4424
HCC1954	0.1349	0	0.0017	0.0693
CAL851	0.3116	0	0.2654	0.0001
HCC38	0.366	0	0.3184	0.3954
DU4475	0.378	0	0.463	0.3671
MCF12A	0.3932	0	0.4401	0.0009
AU565	0.4357	0	0.1025	0.3805
HCC1143	0.4606	0	0.3826	0.0061
MCF7	0	0.0001	0.4122	0.3593
T47D	0	0.03	0.2166	0.4109
MDAMB361	0.0077	0.0357	0.0072	0.4217
MDAMB415	0.0009	0.0663	0.324	0.4321
BT474	0.026	0.1206	0.002	0.1014
EVSAT	0	0.1781	0.3659	0.4796
CAMA1	0.0741	0.2519	0.3151	0
JIMT1	0.2124	0.3299	0.4211	0.0021
BT483	0	0.3611	0.2603	0.3347
MDAMB468	0.4116	0.3684	0.4634	0
ZR751	0.4194	0.3707	0.3085	0.1429
HCC70	0.3912	0.3709	0.4841	0.0724
HDQP1	0.4929	0.3731	0.7233	0.0083
BT20	0.1466	0.3736	0.2018	0
HCC1187	0.3141	0.4089	0.4323	0.0134
MDAMB453	0	0.4091	0.4773	0.3695
HCC1937	0.0808	0.4092	0.459	0.0329
BT549	0.4081	0.4249	0.4408	0.1796
MDAMB436	0.4412	0.4645	0.5501	0.0217

Supplementary Table 08 – Drug sensitivity curves of MDAMB361, HCC38 and AU565 cell-lines: (A) Assayed cell line or background well. In background wells no cells were seeded in ; (B) Biological replicate number; (C) Drug used for treatment or DMSO negative control; (D) Drug concentration or DMSO %. The DMSO % of the negative control was standardized to the DMSO % of the highest drug concentration of each drug treatment; (E) Measured luminescence intensity following 72h treatment. Luminescence level is a measred of cell viability as assessed by Promega CellTiter-Glo® Luminescent Cell Viability Assay and measured by the GloMax® Discover instrument from Promega.

CCL	replicate	treatment	concentration	intensity
HCC38	1	DMSO	0.10%	225400
HCC38	2	DMSO	0.10%	237500
HCC38	3	DMSO	0.10%	228900
HCC38	1	afatinib	0.001µM	122300
HCC38	2	afatinib	0.001µM	136500
HCC38	3	afatinib	0.001µM	131300
HCC38	1	afatinib	0.01µM	193100
HCC38	2	afatinib	0.01µM	199800
HCC38	3	afatinib	0.01µM	198300
HCC38	1	afatinib	0.1µM	216500
HCC38	2	afatinib	0.1µM	221500
HCC38	3	afatinib	0.1µM	201600
HCC38	1	afatinib	1µM	230300
HCC38	2	afatinib	1µM	234900
HCC38	3	afatinib	1µM	216300
HCC38	1	afatinib	4µM	211100
HCC38	2	afatinib	4µM	223100
HCC38	3	afatinib	4µM	208900
background_for_HCC38	1	no	no	1380
background_for_HCC38	2	no	no	2230
background_for_HCC38	3	no	no	1040
AU565	1	DMSO	0.10%	216000
AU565	2	DMSO	0.10%	204500
AU565	3	DMSO	0.10%	205900
AU565	1	afatinib	0.001µM	24600
AU565	2	afatinib	0.001µM	20590
AU565	3	afatinib	0.001µM	20470
AU565	1	afatinib	0.01µM	50620
AU565	2	afatinib	0.01µM	49000
AU565	3	afatinib	0.01µM	45810
AU565	1	afatinib	0.1µM	63440
AU565	2	afatinib	0.1µM	61510
AU565	3	afatinib	0.1µM	56110
AU565	1	afatinib	1µM	80700
AU565	2	afatinib	1µM	79400

AU565	3	afatinib	1µM	81640
AU565	1	afatinib	4µM	165400
AU565	2	afatinib	4µM	174100
AU565	3	afatinib	4µM	162800
background_for_AU565	1	no	no	520
background_for_AU565	2	no	no	620
background_for_AU565	3	no	no	450
MDA-MB-361	1	DMSO	0.10%	168100
MDA-MB-361	2	DMSO	0.10%	155900
MDA-MB-361	3	DMSO	0.10%	146400
MDA-MB-361	1	afatinib	0.001µM	35610
MDA-MB-361	2	afatinib	0.001µM	31690
MDA-MB-361	3	afatinib	0.001µM	32980
MDA-MB-361	1	afatinib	0.01µM	86210
MDA-MB-361	2	afatinib	0.01µM	85490
MDA-MB-361	3	afatinib	0.01µM	78680
MDA-MB-361	1	afatinib	0.1µM	103600
MDA-MB-361	2	afatinib	0.1µM	99920
MDA-MB-361	3	afatinib	0.1µM	96510
MDA-MB-361	1	afatinib	1µM	109300
MDA-MB-361	2	afatinib	1µM	105700
MDA-MB-361	3	afatinib	1µM	109900
MDA-MB-361	1	afatinib	4µM	116900
MDA-MB-361	2	afatinib	4µM	121100
MDA-MB-361	3	afatinib	4µM	119800
background_for_MDA-MB-361	1	no	no	540
background_for_MDA-MB-361	2	no	no	580
background_for_MDA-MB-361	3	no	no	1070

Supplementary Table 09 – Dose response cuvers of HER2+ and HER2- FACS-sorted cell subpopulations of MDAMB361 cell-line against etoposide and afatinib: (A) Status of HER2 expression; (B) Biological replicate number; (C) Drug treatment or DMSO negative control ; (D) Drug concentration or DMSO %. The DMSO % of the negative control was standardized to the DMSO % of the highest drug concentration of each drug treatment; (E) Total number of nuclei counted in the well; (F) nuclei count after filtering for artefacts in the image analysis pipeline.

MDA-MB-361 subpopulation	replicate	treatment	conc.	nuclei count	nuclei count filtered
HER2_negative	1	DMSO (negative control for afatinib treatment)	0.10%	630	452
HER2_negative	2	DMSO (negative control for afatinib treatment)	0.10%	1799	1159
HER2_negative	1	afatinib	4µM	943	643
HER2_negative	2	afatinib	4µM	794	519
HER2_negative	3	afatinib	4µM	799	518
HER2_negative	1	afatinib	1µM	860	543
HER2_negative	2	afatinib	1µM	681	489
HER2_negative	3	afatinib	1µM	579	363
HER2_negative	1	afatinib	0.25µM	2414	1095
HER2_negative	2	afatinib	0.25µM	954	646
HER2_negative	3	afatinib	0.25µM	655	435
HER2_negative	1	afatinib	0.06µM	805	434
HER2_negative	2	afatinib	0.06µM	1018	635
HER2_negative	1	afatinib	0.016µM	464	210
HER2_negative	2	afatinib	0.016µM	440	188
HER2_negative	1	DMSO (negative control for etoposide treatment)	0.10%	1177	785
HER2_negative	2	DMSO (negative control for etoposide treatment)	0.10%	1133	733
HER2_negative	3	DMSO (negative control for etoposide treatment)	0.10%	1151	732
HER2_negative	1	etoposide	40µM	1378	744
HER2_negative	2	etoposide	40µM	717	405
HER2_negative	3	etoposide	40µM	443	316
HER2_negative	1	etoposide	10µM	537	338
HER2_negative	2	etoposide	10µM	394	264
HER2_negative	3	etoposide	10µM	420	218
HER2_negative	1	etoposide	2.5µM	467	207

HER2_negative	2	etoposide	2.5µM	728	447
HER2_negative	3	etoposide	2.5µM	487	267
HER2_negative	1	etoposide	0.6µM	429	236
HER2_negative	2	etoposide	0.6µM	639	339
HER2_negative	3	etoposide	0.6µM	559	287
HER2_negative	1	etoposide	0.16µM	500	185
HER2_negative	2	etoposide	0.16µM	420	219
HER2_negative	3	etoposide	0.16µM	322	116
HER2_positive	1	afatinib	0.016µM	1860	588
HER2_positive	2	afatinib	0.016µM	2037	745
HER2_positive	3	afatinib	0.016µM	2401	920
HER2_positive	1	afatinib	0.06µM	3082	1549
HER2_positive	2	afatinib	0.06µM	4000	1971
HER2_positive	3	afatinib	0.06µM	3703	1791
HER2_positive	1	afatinib	0.25µM	4027	2188
HER2_positive	2	afatinib	0.25µM	3816	1881
HER2_positive	3	afatinib	0.25µM	4309	1886
HER2_positive	1	afatinib	1µM	3899	2086
HER2_positive	2	afatinib	1µM	4009	2176
HER2_positive	3	afatinib	1µM	2620	1375
HER2_positive	1	afatinib	4µM	2314	1261
HER2_positive	2	afatinib	4µM	3392	2005
HER2_positive	3	afatinib	4µM	4171	2219
HER2_positive	1	DMSO (negative control for afatinib treatment)	0.10%	5190	2847
HER2_positive	2	DMSO (negative control for afatinib treatment)	0.10%	4133	2305
HER2_positive	3	DMSO (negative control for afatinib treatment)	0.10%	4009	2196
HER2_positive	1	etoposide	0.16µM	2089	791
HER2_positive	2	etoposide	0.16µM	2609	1124
HER2_positive	3	etoposide	0.16µM	2803	1220
HER2_positive	1	etoposide	0.6µM	2349	965
HER2_positive	2	etoposide	0.6µM	2488	939
HER2_positive	3	etoposide	0.6µM	2891	1265
HER2_positive	1	etoposide	2.5µM	3411	1621
HER2_positive	2	etoposide	2.5µM	3843	2020
HER2_positive	3	etoposide	2.5µM	4281	1797
HER2_positive	1	etoposide	10µM	4658	2541
HER2_positive	2	etoposide	10µM	4025	2267
HER2_positive	3	etoposide	10µM	3842	1408
HER2_positive	1	etoposide	40µM	3472	1966
HER2_positive	2	etoposide	40µM	3998	2359

HER2_positive	3	etoposide	40µM	3607	2073
HER2_positive	1	DMSO (negative control for etoposide treatment)	0.10%	3193	1820
HER2_positive	2	DMSO (negative control for etoposide treatment)	0.10%	3197	1832
HER2_positive	3	DMSO (negative control for etoposide treatment)	0.10%	4250	2311

Supplementary Table 10 – Flow cytometry analysis of HER2 expression in MDAMB361 cell line nrichement following treatment with either afatinib of etoposide . (A) Assayed cell line name, or background well; (B) Biological replicate number; (C) Drug used for treatment or DMSO negative control; (D) Drug concentration or DMSO %; (E) % of mouse anti-human HER2 Ab positive cells measured with flow cytometry.

CCL	replicate	treatment	concentration	%_HER2_positive_cells
MDA-MB-361	1	DMSO	0.10%	72.09%
MDA-MB-361	2	DMSO	0.10%	47.62%
MDA-MB-361	3	DMSO	0.10%	72.50%
MDA-MB-361	4	DMSO	0.10%	66.15%
MDA-MB-361	5	DMSO	0.10%	52.41%
MDA-MB-361	6	DMSO	0.10%	83.29%
MDA-MB-361	1	afatinib	1μM	6.05%
MDA-MB-361	2	afatinib	1μM	21.21%
MDA-MB-361	3	afatinib	1μM	21.13%
MDA-MB-361	4	afatinib	1μM	21.53%
MDA-MB-361	5	afatinib	1μM	46.93%
MDA-MB-361	6	afatinib	1μM	45.05%
MDA-MB-361	1	etoposide	10μM	83.15%
MDA-MB-361	2	etoposide	10μM	95.94%
MDA-MB-361	3	etoposide	10μM	78.02%
MDA-MB-361	4	etoposide	10μM	96.90%
HCC38	1	no	no	2.77%
HCC38	2	no	no	4.84%
HCC38	3	no	no	0.78%
HCC38	4	no	no	4.85%
HCC38	5	no	no	5.48%
HCC38	6	no	no	8.15%
AU565	1	no	no	97.71%
AU565	2	no	no	70.13%
AU565	3	no	no	68.07%
AU565	4	no	no	90.67%
AU565	5	no	no	90.25%
AU565	6	no	no	98.78%

Supplementary Table 11 – MDAMB361 sensitivity to afatinib and etoposide: (A) Assayed cell line name, or background well; (B) Biological replicate number; (C) Drug used for treatment or DMSO negative control; (D) Drug concentration or DMSO %; (E) Measured luminescence intensity following 72h treatment. Luminescence level is a measured of cell viability as assessed by Promega CellTiter-Glo® Luminescent Cell Viability Assay and measured by the GloMax® Discover instrument from Promega.

CCL	replicate	treatment	concentration	luminescence_intensity
MDA-MB-361	1	DMSO	0.10%	134000
MDA-MB-361	2	DMSO	0.10%	133000
MDA-MB-361	3	DMSO	0.10%	167800
MDA-MB-361	1	afatinib	1μM	65370
MDA-MB-361	2	afatinib	1μM	65160
MDA-MB-361	3	afatinib	1μM	64740
MDA-MB-361	1	etoposide	10μM	58500
MDA-MB-361	2	etoposide	10μM	62700
MDA-MB-361	3	etoposide	10μM	62460
background	1	no	no	99
background	2	no	no	109
background	3	no	no	101
